# Multi-Omics Analysis of Low-Temperature Fruiting Highlights the Promising Cultivation Application of the Nutrients Accumulation in *Hypsizygus marmoreus*

**DOI:** 10.3390/jof8070695

**Published:** 2022-06-30

**Authors:** Ming Gong, Tianyu Huang, Yan Li, Jinxin Li, Lihua Tang, Erzheng Su, Gen Zou, Dapeng Bao

**Affiliations:** 1 National Engineering Research Center of Edible Fungi, Key Laboratory of Edible Fungi Resources and Utilization (South), Ministry of Agriculture, Institute of Edible Fungi, Shanghai Academy of Agricultural Sciences, Shanghai 201403, China; lilysearch@163.com (M.G.); swallow2005@live.cn (Y.L.); tanglihua@saas.sh.cn (L.T.); 2 Department of Food Science and Technology, College of Light Industry and Food Engineering, Nanjing Forestry University, Nanjing 210037, China; ezhsu@njfu.edu.cn; 3 College of Food Science and Engineering, Jiangsu Ocean University, Lianyungang 222005, China; htyhty1996@163.com; 4 Research and Development Center, Shanghai Finc Bio-Tech Inc., Shanghai 201401, China; lijinxin82@163.com

**Keywords:** low-temperature fruiting, multi-omics, cultivation, GCN2, *Hypsizygus marmoreus*

## Abstract

*Hypsizygus marmoreus* is a representative edible mushroom with low-temperature fruiting after a long postripening (LFLP). Clarifying the mechanism of LFLP and applying a rigorous low-temperature-limited process will optimize the mushroom cultivation process. This study performed an integrative multi-omics analysis of the molecular mechanism of LFLP in combination with genetic, physiological, and cultivation confirmation. The results showed that the amino acid content was increased during LFLP, mainly arginine. pH analysis showed acidification in the postripening stage and alkalization in the substrates of the reproductive growth stage. An enzyme activity test confirmed the increased enzyme activity of arginase and citrate synthase in the postripening stage. Weighted gene coexpression network analysis of the transcriptome and metabolomics indicated that pH variation is correlated mainly with changes in citrate and arginine. Multi-omics reveals a straightforward way of providing enriched materials for amino acid biosynthesis, namely, synergistically elevating citric acid and arginine through enhanced activity of the arginine synthesis branch pathway in the citrate cycle. Our study confirmed that GCN2 mediated metabolic adaptation by enhancing protein translation, highlighting its regulatory role during LFLP. Exogenously added citric acid and arginine shortened the postripening period by 10 days and increased the fruiting body yield by 10.2~15.5%. This research sheds light on the molecular mechanism of LFLP in *H. marmoreus* and highlights the promising application of nutrient accumulation in high-efficiency cultivation.

## 1. Introduction

The rapidly growing global population has also resulted in growing food demand and increased agricultural output, leading to the generation of agricultural byproducts and wastes, such as sugarcane bagasse, rice husks, cotton stalks, straw, and stover [1]. Mushrooms vegetatively grow on these stubborn residual biomasses and convert them into high-value edible and medicinal products [2,3,4]. In China, the economic output of edible fungi has ranked as the fifth-largest agricultural sector after grain, vegetables, fruit trees, and oilseeds, ahead of tea, sugar, and cotton [5]. Mushroom cultivation must go through vegetative and reproductive growth to form the fruiting body. For most mushrooms, the transition from vegetative to reproductive development must be induced by low-temperature treatment. Low-temperature treatment is a necessary process allowing many mushrooms to enter the reproductive stage [6,7], such as *Lentinus edodes* [8], *Agaricus bisporus* [9], *Flammulina velutipes* [10], *Pleurotus eryngii subsp. tuoliensis* (Bailinggu) [11], *Pleurotus nebrodensis* [11], *Armillaria mella* [12], and *Hypsizygus marmoreus*. In addition, the overgrown mycelia of some mushrooms need to go through a long postripening stage before low-temperature treatment. These processes consume much energy and increase production costs, limiting the cultivation of mushrooms from being low carbon and sustainable agricultural industry. It is essential to unravel the molecular mechanisms behind these transitions to guide sustainable mushroom cultivation.

*Hypsizygus marmoreus*, also known as beech mushroom, crab mushroom, sea mushroom, etc. [13,14], has a month-long postripening stage and a typical low-temperature fruiting stage after a long postripening (LFLP). Premature interruption of the postripening stage for fruiting results in a drastic reduction in production. Furthermore, the subsequent low-temperature treatment of mature mycelia is the main trigger for primordia initiation and plays a crucial role in the mushroom formation and the subsequent yields of *H. marmoreus*. Therefore, it can be used as a typical model organism to study the molecular mechanism of the postripening stage and the low-temperature fruiting of mushrooms.

The regulation of cytosolic mRNA translation is crucial for rapid adaptation to environmental stress conditions. General control nonderepressible 2 (GCN2) is a Ser-Thr kinase found in all eukaryotic organisms that phosphorylate eukaryotic translation initiation factor 2 (eIF2) under cold stress, which inhibits the initiation of protein translation and is essential for cold tolerance in Arabidopsis [15]. Recent research has shown that GCN2 is activated under various stresses, including cold treatments [16]. Therefore, it is desirable to study the regulatory role of GCN2-mediated translation in the LFLP of *H. marmoreus*.

In this study, we investigated the expression profile of the representative developmental stages in *H. marmoreus* using quantitative transcriptome, amino acid (AA), and organic acid-targeted metabolomics to reveal the molecular mechanism of LFLP in *H. marmoreus*. Arginine and citrate could act as postripening biomarkers in *H. marmoreus*. The enhanced GCN2-mediated translation pathway is essential for promoting LFLP in *H. marmoreus*. These findings have the potential to guide lower-cost, low-carbon mushroom cultivation methods.

## 2. Materials and Methods

### 2.1. Sample Preparation

The strain Finc-W-247 is the primary cultivar planted by Shanghai Fengke Science and Technology Co., Ltd., stored in China’s Typical Culture Preservation Center (Wuhan University). The strain was cultured with cottonseed shell 15%, wood chips 25%, rice bran 20%, corn cob 15%, bran 19%, cornmeal 5%, lime 1%, and water content 65%. The evenly stirred medium was put into an 850 mL cultivation bottle for sterilization and inoculation after cooling. The inoculated culture bottles were transferred to the culture chamber for cultivation. The temperature of the culture chamber was generally set at 22–23 °C, the humidity was 70%, and the CO_2_ concentration was 2500–3500 mg/kg. After 30 to 35 days of culture, mycelia were overgrown within the bottle. After approximately 40 days of culture, the mycelia reached physiological maturity (this period is called the postripening stage), and the scratching fungus operation was carried out. Then, the cultivation bottle was transferred to the mushroom room, where the temperature was 14–16 °C, the air humidity was 95–100%, and the CO_2_ concentration was 1500–2500 mg/kg. After approximately 22 days of low-temperature cultivation, fruiting was completed from the primordium stage.

The six growth stages were sampled and stored at −80 °C until testing. They included the overgrown mycelia stage (OG), the postripening culture for 15 d (LR15), the postripening culture for 30 d (LR30), the primordium phase (PS), the droplets phase (with apparent differentiation of the cap and stalk) (DS), and the fruiting body stage (FS). The three samples in the substrates of the developmental stage of the mushroom were collected to understand the metabolic flux of substrates in fruiting stages since metabolites were secreted into the substrates. They include the substrates of the primordium phase (PSR), the substrates of the droplets phase (DSR), and the substrates of the fruiting body stage (FSR).

Finc-W-247 blocks (diameter of 6 mm) were inoculated on potato dextrose agar medium in a 90 mm diameter Petri dish and cultured at 25 °C for 13 days. Then, mycelia were collected and placed at 4 °C for 0, 2, 4, 6, and 8 h and stored at −80 °C until qPCR analysis.

All assays in this study were performed in three independent biological experiments with at least three replicates.

### 2.2. Absolute Quantitative Transcriptome of Nine Developmental Stages of H. marmoreus

The samples of nine developmental stages of *H. marmoreus* were collected and lyophilized, and total RNA was extracted using TRIzol reagent (Invitrogen, Carlsbad, CA, USA) according to the manufacturer’s instructions. There were three biological replicates in this study. RNA extraction, RNA quantity and purity determination, and RNA integrity evaluation were performed according to methods described in previous research [17]. The clean reads were compared to the reference *H. marmoreus* genome [18] using Hisat2 software. PSORTb v3.03 was used for protein subcellular localization prediction [19]. The details about the transcriptome library construction and data analysis can be found in recent studies [20].

### 2.3. Amino Acid-Targeted Metabolome of the Developmental Stages of H. marmoreus

An amino acid-targeted metabolome was used to analyze the metabolites of the nine developmental stages of *H. marmoreus*. There were six biological replicates in this study. LC-MS detection was performed for each standard solution of amino acids and the treated samples. The heatmap program package in R (V3.3.2) was used for agglomerate hierarchical data clustering. The R language ropls package was used for principal component analysis (PCA). The standard for differential metabolites was *p*-value ≤ 0.05 and VIP (variable importance for the projection) ≥ 1. The details about constructing the Mass spectrometric (MS) detection and data analysis can be found in the supplementary material.

### 2.4. Organic Acid-Targeted Metabolome Detection

An organic acid-targeted metabolome was used to analyze the metabolites of nine developmental stages of *H. marmoreus*. There were six biological replicates in this study. Twenty-six organic acid standard substances were weighed. The single standard stock solution was prepared with methanol or water to make a mixed standard. A 30% methanol aqueous solution (containing 0.1% formic acid) was diluted to make a standard working solution, which was stored at 0 °C until LC-MS detection.

The extraction of metabolites was performed according to the methods described in previous research [21]. An ACQUITY UPLC^®^ BEH C18 column (2.1 × 100 mm, 1.7 μm, Waters Inc., Milford, Massachusetts, USA) was used for chromatographic determination. The sample loading volume was 5 μL, the column temperature was 40 °C, and the mobile phases were A-water (containing 0.1% formic acid) and B-methanol water (containing 0.1% formic acid). Multiple response monitoring was used for scanning. Previous research explicitly referred to the chromatographic and mass spectrometric conditions for computer detection [22,23]. The details about the organic acid-targeted metabolome detection can be found in the supplementary material.

### 2.5. Gene Coexpression Network Construction

Weighted correlation network analysis (WGCNA) can find modules of highly correlated genes. Transcriptome data from the postripening stage (PRS) to the substrates of the reproductive growth stage (SRS) were used as input expression data. The organic acid content and pH sample from the PRS to SRS acted as an attribute matrix. WGCNA of the two types of data was used to analyze the critical module of pH variation from the PRS to the SRS.

Transcriptome data from the PRS, the reproductive growth stage (RGS), and SRS were used as input expression data. AA content from the PRS, RGS, and SRS was used as an input attribute matrix. WGCNA of the two types of data was used to analyze the critical module of the change in AA content in LFLP. The WGCNA R software package was used to perform various aspects of weighted correlation network analysis [24]. For details about the steps of WGCNA, refer to a previously published article [25].

### 2.6. qPCR Assays and Western Blot Analysis

Samples of nine developmental stages and the cold-treated samples of *H. marmoreus* were collected for RNA extraction. Details of the qPCR assay are described in a previous study [26]. The primers used for qPCR are listed in Supplementary Table S1. All qPCR assays were performed in three independent experiments. The expression levels of the mRNAs were normalized to Actin-1 and were calculated using the 2^−ΔΔCt^ method [27]. One-way ANOVA followed by the Tukey test for multiple comparisons was conducted using SigmaPlot Version 12.0 to evaluate significant differences among the groups (*p* < 0.05).

Samples from nine developmental stages of *H. marmoreus* were used for total protein extraction. The polyclonal antibody against GCN2 was purchased from Servicebio (catalog no: GB111095). Recombinant protein corresponding to mouse GCN2 was used for the immunization of a rabbit. Western blot analysis was conducted as described previously [28].

### 2.7. Determination of Enzyme Activity

Samples from nine developmental stages of *H. marmoreus* were collected to determine the enzyme activity. The arginase activity of the samples was measured using an arginase activity assay kit (Shanghai Bohu Biological Technology Co., Ltd., Shanghai, China) according to the manufacturer’s protocol. In this procedure, a 1 g sample was mixed with 9 mL phosphate buffer in an ice bath and then centrifuged at 5000 r/min for 15 min. Then, 10 µL supernatant was used for enzyme activity determination. The absorbance change of the system *ΔA* at a wavelength of 450 nm was measured when assaying enzyme activity. *ΔA* is brought into the linear regression equation *(y =* 0.03290 *x* + 0.09103, *R^2^* = 0.9944, where *x* is arginase activity and y is the sample *ΔA value*) for enzyme activity calculation. The sample was diluted five times as directed by the instructions, and the calculated result multiplied by 5 is the actual sample concentration (U/L).

The citrate synthase of the samples was measured using a citrate synthase assay kit (Shanghai Bohu Biological Technology Co., Ltd., Shanghai, China) according to the manufacturer’s protocol. *ΔA* is brought into the linear regression equation (*y =* 0.06232 *x* *+* 0.1375, *R^2^* = 0.9981, where *x* is citrate synthase activity and y is the sample *ΔA value*) for enzyme activity calculation. The sample was diluted five times as directed by the instructions, and the calculated result multiplied by 5 is the actual sample concentration (IU/L).

### 2.8. Cultivation Experiment with Exogenous Arginine and Citric Acid

A 10 mL solution of H_2_O, Arg (6 mmol/L), and citric acid (200 mg/L) was injected into the OG stage. The operation of scratching the fungus was carried out after postripening the culture for 30 d. The cultivation bottle was transferred to the fruiting room after scratching the fungus, and then the weight of the fruiting body per bottle was measured (g/bottle). CK represents the normal postripening culture for 40 d without any solution addition. Each treatment was repeated with five bottles.

The lightness values (L-value) of the wood chips in the cultivation materials were determined by an SC-10 handheld color meter (Shenzhen Sunenshi Technology Co., LTD., China), and the determination time was postripening culture for 8 d, 15 d, 22 d, and 30 d. An appropriate amount of the culture material at the shoulder of the bottle was taken and laid on a 90 mm plate to measure the L-value of the wood chips in the cultivation material. Three bottles were taken from each treatment, and each bottle was measured twice.

## 3. Results

### 3.1. Enhanced Ribosome Pathway for Low-Temperature Fruiting

**Figure 1:**
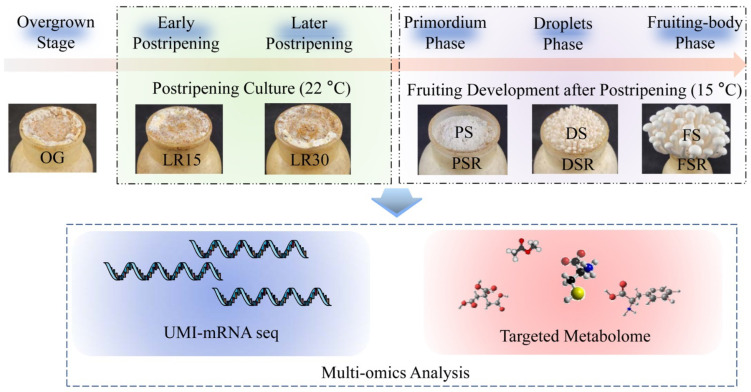
Flow chart of this study. Multi-omics analysis was used to analyze the gene and metabolite expression profiles of *H. marmoreus* at developmental stages to reveal the molecular mechanism of LFLP.

We obtained samples of the different developmental stages of *H. marmoreus* (Figure 1). High-throughput analysis of these samples makes it possible to obtain critical metabolic pathways, amino acids, and organic acids involved in LFLP through multi-omics analysis. The molecular mechanism of LFLP could be further identified in combination with genetic and physiological confirmation (Figure 1). These studies will help to obtain molecular biomarkers of LFLP.

The unique molecular identifier (UMI) absolute quantitative transcriptome of *H. marmoreus* was determined through RNA extraction, purification, detection, library construction, and sequencing. PCA showed that the samples had excellent repeatability and discrimination (Supplementary Figure S1A). Heatmap analysis showed that the samples from the postripening stage (PRS) were clustered together, while the samples from the reproductive growth stage were grouped into another cluster (Supplementary Figure S1B). Venn analysis showed 234 differentially overlapped genes among the samples of the development stages (Supplementary Figure S1C).

There were five significantly enriched pathways (SEPs) in OG vs. PS using the DEGs for which the ratio of upregulated DEGs to downregulated DEGs was greater than 3 and the *p*-value < 0.05. Heatmap analysis showed that ribosomes were the most SEPs in OG vs. PS (Supplementary Figure S1D), indicating the enhanced activity of ribosomes in LFLP. There were no SEPs in OG vs. PS using the DEGs for which the ratio of downregulated DEGs to upregulated DEGs was greater than 3 and the *p*-value < 0.05. Heatmap analysis showed the upregulated ribosomal pathway in PS and DS (Supplementary Figure S2). KEGG enrichment analysis showed that the DEGs in LR30_vs_PS were enriched in the ribosome (Supplementary Figure S3). The ratio of upregulated DEGs to downregulated DEGs in LR30_vs_PS was 28.5 in the enriched ribosome pathway, representing the enhanced ribosome pathway in PS. For the comparison, KEGG enrichment analysis showed that the upregulated DEGs in OG_vs_LR30 were enriched in valine, leucine, and isoleucine degradation, methane metabolism, and glycolysis/gluconeogenesis instead of the ribosome (Supplementary Figure S4). These results indicate that enhanced activity of the ribosome pathway is essential for LFLP in *H. marmoreus*.

### 3.2. Accumulation of Dissociative Amino Acids for Low-Temperature Fruiting

The amino acid metabolism pathway showed an apparent difference in gene expression in the LFLP in *H. marmoreus* (Supplementary Figure S5A). The upregulated DEGs occurring only in the postripening stage were enriched in the cellular amino acid metabolic and biosynthetic processes (Supplementary Figure S5B). The upregulated DEGs in the reproductive growth stage (RGS) were enriched in the alpha-amino acid biosynthetic process (Supplementary Figure S5C). These results indicate that gene transcription is not restricted to the protein translation pathway in the postripening stage. Considering the enhanced activity of the ribosome pathway in the primordium phase instead of the postripening stage, protein translation is inhibited in the postripening stage. This result shifts our attention to the variation in AA content in the LFLP.

**Figure 2:**
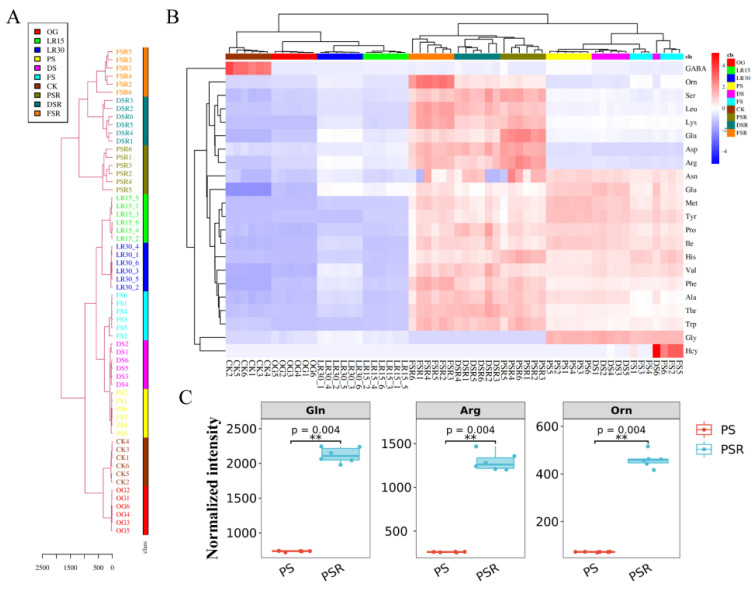
AAs targeted metabolomics of *H. marmoreus* at different developmental stages. (**A**) Hierarchical cluster of AAs. (**B**) Targeted profiling of the AA metabolome of *H. marmoreus* at different developmental stages. (**C**) Statistical tests of the top three AAs with the highest concentrations. ** represents *p* < 0.001.

The AAs in 54 samples (6 samples per development stage or its substrates) in LFLP were detected by LC-MS/MS to obtain the metabolic flow of AAs. The hierarchical cluster of AAs showed that the substrates of the reproductive growth stage (SRS), including PSR, DSR, and FSR, were grouped and separated from the large cluster of the other developmental stages (Figure 2A). Targeted profiling of the AA metabolome showed an apparent increase in AA content in the RGS and SRS (Figure 2B). Statistical tests confirmed the significant upregulation (*p* < 0.001) of several amino acids, such as glutamine (Gln), arginine (Arg), and ornithine (Orn) (Figure 2C). These results indicated that the enriched AAs in RGS and SRS are essential for LFLP.

Analysis of the AA content showed an apparent increase in the SRS (Supplementary Figure S6A). Gln, Arg, and lysine (Lys) exhibited the highest concentrations in the PSR, and Orn showed the highest concentrations in the FSR (Supplementary Figure S6B). PCA of the AAs in the developmental stages of *H. marmoreus* showed that Gln, Arg, Lys, Leucine (Leu), and Orn were separated from the other AAs (Supplementary Figure S7), suggesting that these AAs might play a critical role in the LFLP.

### 3.3. Formation of Arginine Pool Promotes Low-Temperature Fruiting

**Figure 3:**
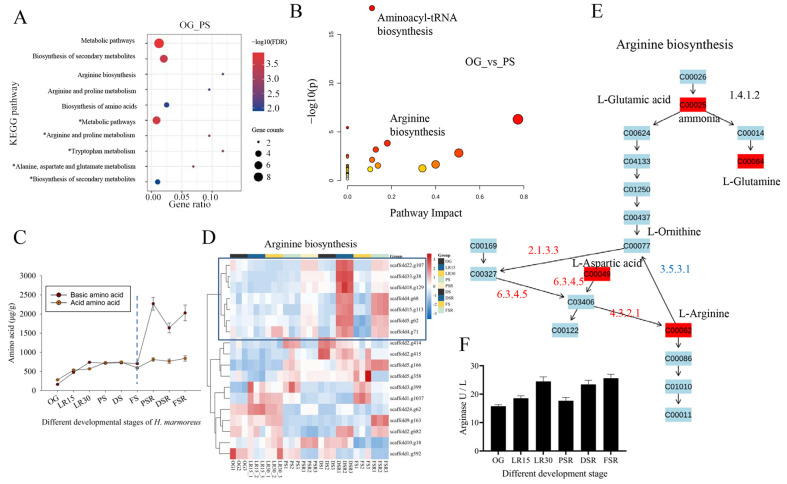
Transcriptome analysis of arginine biosynthesis in *H. marmoreus.* (**A**) KEGG enrichment analysis of the associated differentially expressed genes (DEGs) in OG vs. PS. * represents downregulated DEGs. (**B**) Correlation pathway analysis of the significantly different AAs (SDAs) in OG vs. PS (Impact > 1). (**C**) Analysis of the content of AAs in *H. marmoreus* at different developmental stages. (**D**) Heatmap analysis of gene expression in arginine biosynthesis. (**E**) KEGG pathway view (ko00350) of arginine biosynthesis. Red indicates upregulation in OG vs. PS. Blue suggests upregulation in OG vs. PS. (**F**) Determination of arginase enzyme activity in *H. marmoreus* at different developmental stages.

Correlation analysis of the significant differential AAs (SDAs) and DEGs in OG vs. PS was conducted to obtain the associated DEGs. The associated AAs showed that glutamic acid (Glu), methionine (Met), and glycine (Gly) were the top three AAs with the highest number of associated DEGs (Supplementary Table S3). The associated AAs showed that Glu, aspartic acid (Asp), and Gly were the top three AAs with the highest associated pathways (Supplementary Table S3). The EC annotations of these associated genes showed that N-methyltransferase, N-acetyltransferase, and branched-chain amino acid aminotransferase were the top three enzymes with the most significant number (Supplementary Table S4). KEGG enrichment analysis showed that the associated upregulated DEGs were enriched in arginine biosynthesis and arginine and proline metabolism (Figure 3A). The associated downregulated DEGs were enriched in alanine, aspartate, and glutamate metabolism and tryptophan metabolism. A total of 10 pathways were screened by correlative pathway analysis of the associated SDMs in OG vs. PS (Impact > 1), such as aminoacyl-tRNA biosynthesis and arginine biosynthesis (Figure 3B). The citrate cycle (TCA cycle) (map00020) showed that alanine, aspartate, and glutamate metabolism provided metabolites for downstream arginine biosynthesis. Together with the strong association of Glu and Asp with DEGs and pathways (Supplementary Tables S3 and S4), these observations indicated a critical role of Arg in LFLP. Statistics of the AA content showed the similarity between the acid AAs and the basic AAs in the PRS and RGS (Figure 3C). The content of basic AAs showed a significant increase in SRS relative to acid AAs (Figure 3C). These observations suggest that the substrates of the reproductive growth stage are the Arg pool for supplying the AAs to the reproductive growth.

The absolute quantitative transcriptome was used to obtain SRS expression profiles. PCA showed that all samples were divided into three groups (Supplementary Figure S8A), including the PRS, RGS, and SRS. Heatmap analysis of the expression profile showed apparent differences in the PRS, RGS, and SRS (Supplementary Figure S8B). KEGG enrichment analysis showed decreased activity of the citrate cycle (TCA cycle) compared to the enhanced activity of the pentose phosphate pathway and pentose and glucuronate interconversions in LR30_vs_PSR (Supplementary Table S2). The enhanced activity in the latter two ways could provide large 5-carbon skeletons for the accelerated transformation from 5-carbon to 4-carbon and 7-carbon, producing enriched materials for amino acid biosynthesis.

The upregulated DEGs in PSR_vs_DSR were enriched in arginine biosynthesis (Supplementary Figure S9), indicating the enhanced activity of arginine biosynthesis in SRS and its essential role in LFLP. Homologous genes of arginine biosynthesis in *H. marmoreus* were obtained using Blastp (1 × 10^−30^). Heatmap analysis showed that the upregulated genes in arginine biosynthesis in SRS were clustered and marked by a box (Figure 3D). The KEGG pathway view (ko00350) showed that argininosuccinate synthase (scaffold5.g62) and argininosuccinate lyase (scaffold4.g71) in the box cluster were responsible for the biosynthesis of arginine (Figure 3D,E), indicating the enhanced activity of arginine biosynthesis in SRS instead of PRS. Arginase (3.5.3.1) is an enzyme that catalyzes the hydrolysis of L-arginine into L-ornithine and urea. The increased arginase in the PRS means more hydrolysis of L-arginine, leading to a lower Arg content (Figure 3D). Heatmap analysis showed the increased expression of arginase (scaffold9.g163) in the PRS (Figure 3D and Supplementary Table S5). Enzyme activity tests further confirmed the increased arginase activity in the PRS, consistent with the low concentration of Arg in the PRS (Figure 3F). Analysis of the pH values showed acidification in PRS and subsequent alkalization in SRS (Supplementary Figure S10). The apparent pH change confirms the redirection of nitrogen metabolism to the synthesis of arginine, which agrees with the essential role of the Arg pool for LFLP.

### 3.4. Accumulated Citric Acid and Arginine Promote Amino Acid Biosynthesis

**Figure 4:**
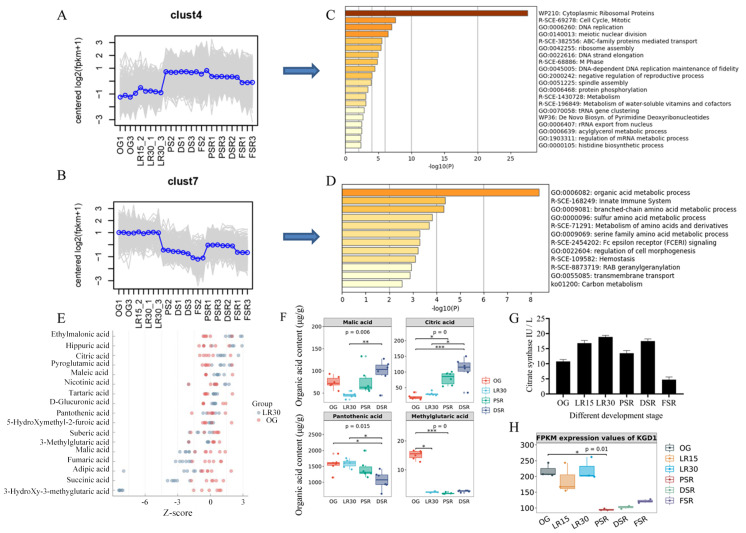
Organic acid-targeted metabolome detection of *H. marmoreus*. (**A**) Trend analysis of DEGs in Cluster 4. (**B**) Trend analysis of DEGs in Cluster 7. (**C**) Enrichment of DEGs in Cluster 4. (**D**) Enrichment of DEGs in Cluster 7. (**E**) Z score results of the content of organic acids in OG_vs_LR30. (**F**) Difference test box diagram of the four top organic acids (>10 μg/g). (**G**) Determination of citrate synthase enzyme activity in *H. marmoreus* at different developmental stages. (**H**) FPKM (fragments per kilobase million) expression values of KGD1 in *H. marmoreus* at different developmental stages. * represents *p* < 0.05; ** represents *p* < 0.01; *** represents *p* < 0.001. to the (**F**) and * represents *p* < 0.05. to the (**H**).

The trend analysis of DEGs showed two representative clusters, Cluster 4 and Cluster 7 (Figure 4A,B). The trends in Cluster 4 showed increased gene expression in RGS and SRS but not PRS. Enrichment of DEGs in Cluster 4 showed that cytoplasmic ribosomal proteins were the most enriched terms (Figure 4C), indicating that protein translation was essential for LFLP. The trends in Cluster 7 showed increased gene expression in the PRS instead of the RGS and SRS (Figure 4B). Enrichment of DEGs in Cluster 7 showed that the organic acid metabolic process was the most enriched pathway (Figure 4D), indicating that organic acid metabolic processes play an important role in LFLP. Heatmap analysis further indicated the enhanced activity of citrate synthesis according to the upregulated CIT2, PYC2, PDB1, and ACO1 in the PRS (Supplementary Figure S11).

Considering the acidification of the postripening stage (Supplementary Figure S10), we focused on the change in organic acids using organic acid-targeted metabolome detection. Heatmap analysis of organic acids showed increased organic acids in the PSR and DSR (Supplementary Figure S12). The Z score of organic acids showed a decreased content in OG_vs_LR30, including 3-hydroxy-3-methylglutaric acid, succinic acid, and malic acid (Figure 4E). These organic acids belong mainly to the downstream products in the citrate cycle. As an upstream metabolite in the citrate cycle, citric acid showed an increase in OG_vs_LR30 (Figure 4E). The difference test box diagram confirmed the continuous apparent upregulation of citric acid in the LFLP (Figure 4F), indicating its critical role in LFLP. Expression analysis showed increased expression of citrate synthase (scaffold4.g43) in the PRS (Supplementary Figure S11). An enzyme activity test further confirmed the increasing activity of citrate synthase in PRS (Figure 4G).

Since citric acid is highly acidic (pKa1 = 3.14 Ka), the upregulated citric acid could explain the acidification in the postripening stage. The difference test box diagram of organic acids showed the obvious downregulation of pantothenic acid in the PSR and DSR (Figure 4F). Based on the high concentrations of pantothenic acid, the downregulated pantothenic acid (pKa = 4.30 ± 0.10) and the increased Arg in the SRS could explain the alkalization in the SRS. The citrate cycle in KEGG showed that citrate provided the materials for the branch pathway of arginine biosynthesis through downstream 2-oxo-glutarate. The downregulated expression of the 2-oxo-glutarate metabolic enzyme (Figure 4H), alpha-ketoglutarate dehydrogenase KGD1 [EC:1.2.4.2] (scaffold6.g76), reduced the activity of the citrate cycle and shifted the branch pathway of arginine biosynthesis in SRS. The synergistically elevated citric acid and arginine reveal a straightforward way of providing enrichment of materials for amino acid biosynthesis.

### 3.5. GCN2-Mediated Translation Regulates Low-Temperature Fruiting

The large AA materials in PSR shifted our attention to translation regulation during reproductive growth. WGCNA of the AA metabolome and transcriptome from the PRS, RGS, and SRS showed 16 modules, among which pink modules correlated with most amino acid (AAs) (Supplementary Figure S13A,B). The total association score of AAs and modules showed that pink modules had the highest score (13.98). Heatmap analysis showed the prominent upregulation of the expression of the hub genes in the pink modules in the DSR and FSR (Supplementary Figure S13C,D). Enrichment analysis of the genes in the pink modules showed the most enrichment of the association of TriC/CCT with the target proteins during biosynthesis (Supplementary Figure S13E) [29], which indicated that regulation of protein biosynthesis is essential in the LFLP of *H. marmoreus*.

**Figure 5:**
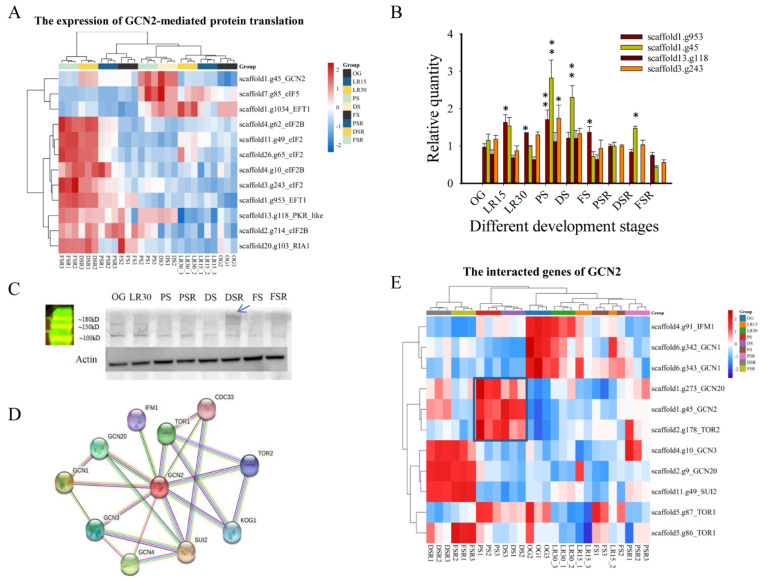
GCN2-mediated translation regulation in low-temperature fruiting after long postripening (LFLP) in *H. marmoreus*. (**A**) Heatmap analysis of key genes in GCN2-mediated translation control in *H. marmoreus* at different developmental stages. (**B**) qPCR analysis of GCN2 and eIF2 in LFLP of *H. marmoreus.* * indicates *p* < 0.05; ** indicates *p* < 0.001. (**C**) Western blot experiment of GCN2 in LFLP. Arrow: the enriched GCN2 with expected MW 160 kD. (**D**) Analysis of GCN2-interacting genes. (**E**) Heatmap analysis of the interacting genes of GCN2.

Considering the feature of low-temperature fruiting of *H. marmoreus*, we focused on the control of translation via the eukaryotic translation initiation factor 2 (eIF2), while four different protein kinases (GCN2, PKR, PEK, HRI) phosphorylate the α subunit of eIF2 in response to various environmental stresses, including cold stress [30]. The key genes’ homologous genes in eIF2 translation control in *H. marmoreus* were obtained using Blastp (1 × 10^−15^). The BLASTP results showed that scaffold1.g45 was the homologous gene with the best hits among the three kinases (GCN2, PEK, and HRI), and scaffold13.g118 was the homologous gene with the best hits of PKR (1.19 × 10^−22^). The Blastp e-values of GCN2, PEK, and HRI to scaffold1.g45 were 5.02 × 10^−111^, 3.96 × 10^−21^, and 4.46 × 10^−32^, respectively, indicating that the conserved GCN2 (scaffold1.g45) was an ancient type of kinase involved in response to different environmental stresses. Heatmap analysis showed the upregulated expression of GCN2 and translation control genes, such as eIF2, eIF2B, and EFT1, in the reproductive growth PS, DS, and DSR (Figure 5A). qPCR confirmed the obviously upregulated expression of GCN2 and eIF2 (*p* < 0.05) in PS compared to OG (Figure 5B). Notably, GCN2 showed obviously (*p* < 0.05) upregulated expression in PS, DSR, and DS compared to OG, indicating its primary role in LFLP (Figure 5B). A western blot experiment with GCN2 showed approximately 160 kD bands enriched in DSR (Figure 5C). The molecular weight of GCN2 is 162.77 kDa, and it falls in this range. qPCR experiments further confirmed that cold stress (4 °C) stimulated the obviously upregulated expression of GCN2 (scaffold1.g45) in *H. marmoreus* mycelia (Supplementary Figure S14). Analysis of genes interacting with GCN2 showed many interacting genes, including GCN20 and TOR2 (Figure 5D). Heatmap analysis showed the upregulated expression of GCN2, GCN20, and TOR2 in PS and DS, and they consisted of a cluster (Figure 5E). These results indicated that low temperature stimulated the GCN2-mediated translation pathway to promote LFLPs.

### 3.6. Cultivation Optimization with Citric Acid and Arginine Addition

**Figure 6:**
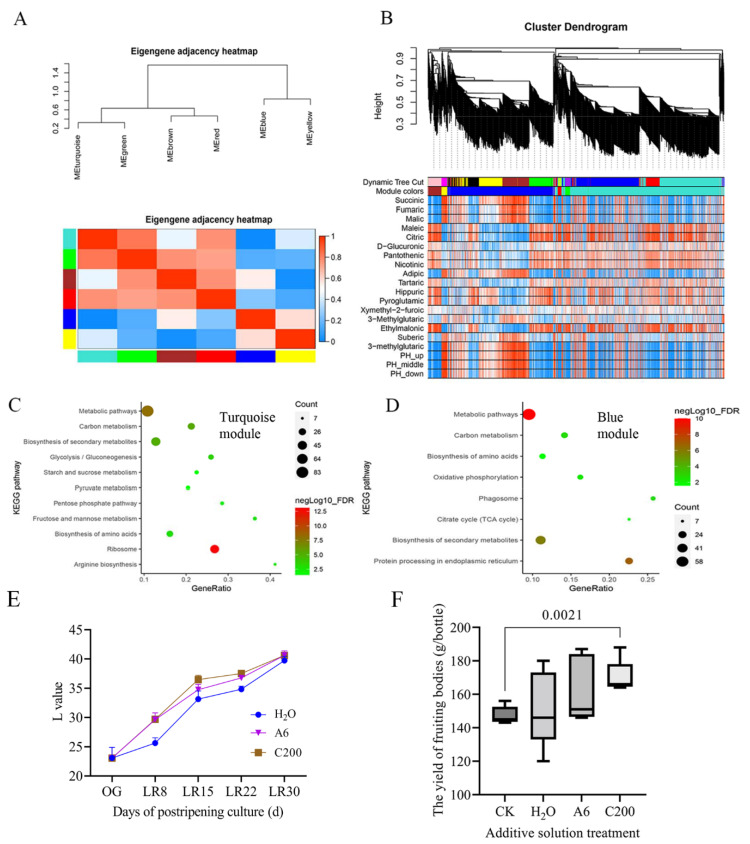
Cultivation experiment with arginine and citric acid addition. (**A**) Clustering dendrograms of module eigengenes and heatmap of eigengene adjacency. Each row and column corresponds to one eigengene within the heatmap. Red indicates high adjacency (positive correlation) within the heatmap, and turquoise indicates low adjacency (negative correlation), as shown by the color legend. (**B**) Hierarchical cluster dendrogram showing coexpressed modules and module trait heatmap. Each leaf on the tree represents a gene. Each colored row indicates a color-coded module that contains a group of highly interconnected genes. (**C**) The KEGG enrichment of genes in turquoise modules. (**D**) The KEGG enrichment of genes in blue modules. (**E**) Change in the L-value of wood chips in the postripening culture under different treatments; H_2_O represents 10 mL H_2_O solution. A6 represents 10 mL arginine solution with 6 mmol/L; C200 represents 10 mL citrate solution with 200 mg/L. (**F**) Statistics of fruiting body yield per bottle in the postripening culture under different treatments. CK represents the traditional postripening culture without any solution addition for 40 days. The illustrations of other treatments can be found in (**E**).

Weighted correlation network analysis (WGCNA) of the organic acid-targeted metabolome and transcriptome from the PRS and SRS showed six modules: blue, brown, green, gray, red, turquoise, and yellow (Figure 6A). The cluster dendrogram showed that the turquoise and blue modules were the two largest modules (Figure 6B). The genes in the turquoise modules were correlated mainly with organic acids, including citric acid and malic acid, while the genes in the blue modules were primarily associated with the pH values (Figure 6B). The genes in the turquoise modules were enriched in the ribosome, arginine biosynthesis, pyruvate metabolism, and biosynthesis of amino acids (Figure 6C). The genes in the blue modules were enriched in protein processing in the endoplasmic reticulum, citrate cycle (TCA cycle), phagosome, and biosynthesis of amino acids (Figure 6D). These observations further indicated that the pH variation in the PRS and SRS was correlated mainly with the changes in arginine and citrate.

The cultivation results showed that the L-value of cultivation substrates supplemented with additive citric acid and arginine on postripening culture for 8 d, 15 d, and 24 d was higher than those supplemented with H_2_O (Figure 6E). Fruiting experiments confirmed that the fruiting body yield of the citric acid treatment (postripening culture for 30 d) was significantly higher than that of CK (postripening culture for 40 d) (*p* = 0.0021) (Figure 6F). The average fruiting body yield per bottle under the citric acid treatment and Arg treatment was 15.468% and 10.176% higher than that under CK. The average fruiting body yield per bottle under the citric acid and Arg treatments was 12.27% and 7.12% higher than the control, respectively. These results confirmed that citric acid and Arg addition could shorten the postripening culture by 10 d and increase the production by at least 10% compared to CK. These observations confirmed that the accumulation of arginine and citrate could promote the fruiting efficiency of *H. marmoreus*.

## 4. Discussion

Vernalization accelerates plant development from the vegetative to the reproductive stage, leading to considerable changes in the metabolite profiles of plants [31]. This study also confirmed that the vernalization-like low-temperature treatment process in mushrooms has similar metabolic changes. Some of these metabolites can be used as biomarkers in mushroom factory culture to optimize the cultivation process. Compared to RGS, the abundant AAs in SRS indicated that SRS develops an AAs pool. Arg (one of the major biomarkers) is the dominant component of the AA pool in SRS (Figure 2B), indicating its essential role in LFLP. The arginine pool plays an essential role in loblolly pine seedlings without prior conversion [32], suggesting that the newly synthesized Arg in SRS is transported rapidly and efficiently for fruiting development without prior conversion. The increased Arg content in SRS indicates that Arg is synthesized mainly in SRS. The pH value of the upper substrates in the cultivation bottle was lower than that in the lower part in LR15 (Supplementary Figure S10), compared to the opposite trend of pH value in DSR. Our results showed the opposite trend of pH change in the spatial distribution inner the cultivation bottle in LFLP (Supplementary Figure S10), suggesting an upwards metabolic flux of arginine for supplying the fruiting.

Arginase (scaffold9.g163) showed high gene expression in the postripening stage (Figure 4B,D), which supports the increased enzyme activity of arginase in the postripening stage (Figure 4E). The absence of arginase during the initiation of fruit growth indicated a redirection of nitrogen metabolism to the synthesis of arginine [33]. The lower arginase activity in the PSR stage (Figure 4E) explained the increased content of Arg in SRS, suggesting a redirection of nitrogen metabolism in the PRS to arginine synthesis in SRS. The addition of Arg in the OG stage promoted fruiting efficiency (Figure 6F), indicating that enhanced nitrogen metabolism activity is essential for postripening. This result agrees with the developmental velocity influenced by the external addition of arginine to the ascomycete *Sordaria macrospora* Auersw [34].

Another biomarker found in this study is citrate, which has a high concentration in the PSR. In contrast, citrate synthase enzyme activity decreased in this stage. Previous research reported that a mitochondrial methyltransferase inhibited citrate synthase activity through metabolite-sensitive lysine methylation [35]. Multi-omics analysis showed that methyltransferase was the top associated enzyme with the most significant number in OG vs. PS (Figure 3B). Subcellular localization prediction indicated that two methyltransferases (scaffold27.g50 and scaffold2.g228) were located in mitochondria, and they showed increased expression in the postripening stage, suggesting that methylation also plays an inhibitory role against citrate synthase activity in the postripening stage. The methylation level might be reduced due to the decreased expression of methyltransferases in the low-temperature treatment, which is consistent with a previous study that showed that cold treatment selectively decreased the methylation level in the root of maize [36]. The regular citrate synthase activity may account for the enriched citrate in the PSR. The increased citrate is positively correlated with the high AA content in the LFLP of *H. marmoreus,* which is consistent with the finding that increased citrate may induce AA synthesis in citrus fruit [37]. A high concentration of citrate might inhibit the activity of the citrate cycle and shift it to the branch AA synthesis pathways.

**Figure 7:**
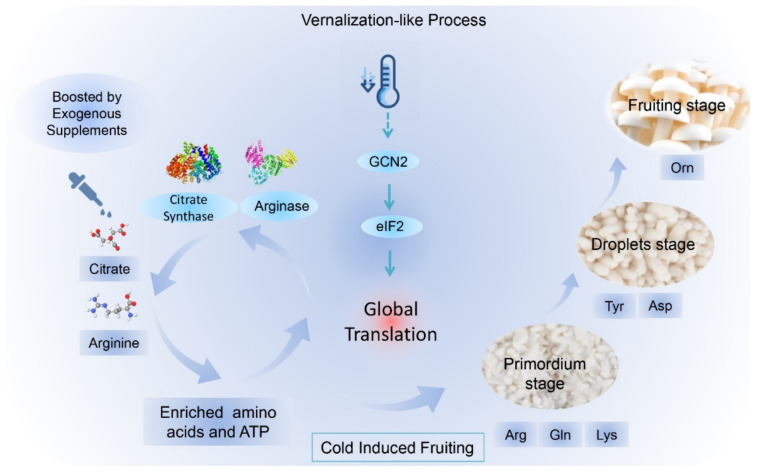
A potential model of GCN2-mediated translation regulation for low-temperature fruiting after long postripening (LFLP) in *H. marmoreus*. Low temperature stimulated the GCN2-mediated eIF2 translation pathway with the enriched amino acids and ATP in LFLP, which are essential for promoting global translation. The synergistically increased citrate and Arg may promote amino acid synthesis and ATP production for global translation in *H. marmoreus*.

A close link between the upregulation of low-temperature-associated proteins and vernalization fulfillment in wheat [38] suggests a role of cold stress-responsive genes in the vernalization-like process in *H. marmoreus*. GCN2 is a serine/threonine-protein kinase that regulates translation in response to stressors such as amino acid and purine deprivation, cold shock, wounding, cadmium exposure, and UV-C exposure [39]. In yeast and animals, phosphorylation of the α-subunit of eIF2 is the most thoroughly characterized event regulating global translation under stress. GCN2 is activated under various stresses, including cold treatments [16]. GCN2 and eIF2 were upregulated under low-temperature fruiting (Figure 5B), indicating that low temperature stimulated the GCN2-mediated eIF2 pathway to promote global protein translation (Figure 7). Activated GCN2 phosphorylates the α-subunit of eIF2, drastically inhibiting protein synthesis in most species [39]. However, GCN2-mediated protein translation in *H. marmoreus* promotes the activation of protein synthesis, and this constitutes a significant exception; it also occurs in wheat under cold shock [40]. These results suggest that the stress response mechanism via GCN2-mediated eIF2α phosphorylation is not identical in all eukaryotes [40].

Protein translation is controlled by versatile ATP-driven machines [41], which means that enriched ATP is essential for global protein translation. A high concentration of citrate means the accumulation of ATP due to the enhanced activity of the citrate cycle during the postripening process of mycelia. Reduced activity of the citrate cycle means a shift to the branch AA synthesis pathways. Our observations reveal a straightforward way of providing enrichment materials for amino acid biosynthesis, namely, synergistically elevated citric acid and arginine levels. The downregulated expression of the enzyme KGD1 (Figure 4H) indicates the reduced activity of the downstream pathway of the citrate cycle. The accumulation of citric acids could provide enriched materials to the arginine synthesis branch pathway, promoting arginine accumulation. The accumulation of arginine is necessary for protein translation in the LTLP of *H. marmoreus*, which is consistent with previous studies showing that sufficient arginine concentrations are required for normal protein accumulation rates [42]. Our model suggested that the synergistically rising citrate and arginine induced amino acid synthesis and ATP production for global translation (Figure 7). The enhanced activity of the GCN2-mediated eIF2 translation pathway promoted global protein translation in the LFLP of *H. marmoreus* with enriched AAs and ATP (Figure 7). The analysis of this process contributes to a low-cost mushroom cultivation process and provides a breeding direction for mushrooms suitable for sustainable cultivation.

In addition to the pivotal cold induction, other factors are also involved in forming fruiting bodies. H_2_O addition positively affected the yield of the fruiting body compared to CK (Figure 6F). The decreased CO_2_ concentration during the fruiting of *H. marmoreus* may agree with the increased respiration activity during primordia formation in the development of fruiting bodies [43]. Future research needs to integrate these factors to reveal this complex process’s mechanism further.

## 5. Conclusions

Our findings reveal that protein translation is essential for the LFLP in *H. marmoreus* with the synergistic accumulation of citric acid and arginine. The highly accumulated concentration of citrate might inhibit the activity of the citrate cycle and shift it to the branch arginine synthesis pathways. Low temperature stimulated GCN2-mediated global protein translation control in LFLPs with enriched ATP and AAs. The SRS acts as the AAs pool for supplying the AAs for the LFLP in *H. marmoreus*. Citric acid and arginine additions could increase fruiting efficiency in *H. marmoreus* and reduce the cost of large-scale cultivation. The increasing activity of citrate synthase and arginase could be used as screening criteria for superior breeding varieties. More importantly, our pilot study of LFLP in *H. marmoreus* contributes to a better understanding of the physiological and genetic mechanisms involved in fungal growth and development.

## Data Availability

The data supporting the findings of this work are available within the paper and its supplementary files. The data sets generated and analyzed during this study are available from the corresponding author upon request.

## Supplementary Materials

The following supporting information can be downloaded at: https://www.mdpi.com/article/10.3390/jof8070695/s1, Figure S1: Unique molecular identifier (UMI) absolute quantitative transcriptome analysis of developmental stages of *H. marmoreus*; Figure S2: Heatmap analysis of gene expression in the ribosomal pathways; Figure S3: KEGG enrichment analysis of the DEGs in LR30_vs_PS; Figure S4: KEGG enrichment analysis of the DEGs in OG vs. LR30; Figure S5: Expression profile of the amino acid metabolism pathway in low-temperature fruiting after long postripening (LFLP) in *H. marmoreus*; Figure S6: Targeted profiling of the amino acid metabolome of different developmental stages of *H. marmoreus*; Figure S7: PCA of the AAs in the developmental stages of *H. marmoreus*; Figure S8: UMI absolute quantitative transcriptome analysis of the developmental stages of *H. marmoreus*, including the substrates of the reproductive growth stage (SRG); Figure S9: KEGG enrichment analysis of the DEGs in PSR vs. DSR; Figure S10: pH value of substrates in the developmental stages of *H. marmoreus*. The pH values of the substrates from the upper, middle, and lower parts of the cultivation bottle were measured; Figure S11: Heatmap analysis of the expression of citrate cycle in the developmental stages of *H. marmoreus*; Figure S12: Heatmap analysis of organic acids in the postripening stage (PRS) and SRG; Figure S13: WGCNA of the AA-targeted metabolome and transcriptome. (A) Hierarchical cluster dendrogram showing coexpressed modules and a module trait heatmap. Each leaf on the tree represents a gene. Each coloured row indicates a colour-coded module that contains a group of highly interconnected genes. (B) Heatmap showing the correlation between the modules and AAs. Each row corresponds to a module, whereas each column corresponds to an AA. The correlation coefficient between a given module and an AA is indicated by the colour of the cell at the row-column intersection. Blue and red indicate positive and negative correlations, respectively. (C) The hub genes in the pink modules. (D) Heatmap of the hub genes in the pink module. (E) Enrichment analysis of the genes in the pink modules; Figure S14: qPCR analysis of GCN2 and eIF2 in the 4 °C cold stress experiment of *H. marmoreus* mycelia. Table S1: Primers used for qPCR; Table S2: KEGG enrichment analysis of the DEGs in LR30 vs. PSR; Table S3: Correlation analysis of differentially expressed genes (DEGs) and significantly different AAs (SDAs) in OG vs. PS. The associated AAs obtained by correlation analysis of DEGs and SDAs; Table S4: The associated enzymes obtained by correlation analysis of DEGs and SDAs; Table S5 KEGG annotation of arginine biosynthesis.
