# Supplementary material for: Multi-Omics Analysis of Low-Temperature Fruiting Highlights the Promising Cultivation Application of the Nutrients Accumulation in Hypsizygus marmoreus
Source: J Fungi (Basel). 2022 Jun 30;8(7):695. doi: 10.3390/jof8070695 (PMC9315786; doi:10.3390/jof8070695)
Supplement: Supplementary file 1 [file jof-08-00695-s001.zip › jof-1754506-supplementary.pdf]

Supplementary material

Amino acid-targeted metabolome of the developmental stages of H. marmoreus

An amino acid-targeted metabolome was used to analyse the metabolites of the nine developmental stages of *H. marmoreus*. There were six biological replicates in this study. A 15 mg tissue sample was transferred into a 2 mL EP tube with 200 μL 10% formic acid methanol solution-ddH_2O (1:1. VVV) solution and 50 mg glass beads. Then, the mixed sample was put into the high-throughput tissue grinding machine and shaken at 60 Hz for 1 min, which was repeated twice. Thirty microlitres of supernatant were obtained and then added to 120 μL of 10% formic acid-methanol solution-ddH_2O (1:1. VVV) and vortexed for 30 s. One hundred microlitres of the diluted sample were obtained, and then 100 μL of 100 ppb dual-isotope internal standard was added and vortexed for 30 s. The supernatant was filtered through a 0.22 μm membrane, and the collected filtrate was added to the test bottle.

Chromatographic separation was accomplished in a Waters ACQUITY UPLC. Chromatographic conditions: chromatography column Agilent HP-INNOWAX capillary column (30 m × 0.25 mm ID × 0.25 μm); shunt injection, injection volume 1 μL, shunt ratio 10:1, injector temperature 250 °C; ion source temperature 230 °C; transmission line temperature 250 °C; quadrupole temperature 150 °C. The temperature program started at 90 °C; then, the temperature was heated to 120 °C at 10 °C/min. Then, the temperature was heated to 150 °C at 5 °C /min. Finally, the temperature was heated to 250 °C at 25 °C /min for 2 min. The carrier gas was helium, and the carrier gas flow rate was 1.0 mL/min. Mass spectrometric (MS) detection was executed on an AB 4000 triple quadrupole mass spectrometer with an electrospray ionization (ESI) source and positive ion ionization mode. The ion source temperature was 500 °C, the ion source voltage was 5500 V, the collision gas was 6 psi, the curtain gas was 30 psi, and the atomization gas and auxiliary gas were 50 psi. Multiple response monitoring (MRM) was used for scanning.

The appropriate amount of 22 kinds of standard amino acids was weighed and dissolved in methanol or water to prepare a single standard mother liquor. A proper amount of each mother liquor was taken to make a mixed standard, diluted with water to the appropriate concentration to obtain a standard solution. LC–MS detection was performed for each standard solution and the treated samples. Shanghai Personal Biotechnology Co. implemented the LC–MS detection. The pheatmap program package in R (V3.3.2) was used for agglomerate hierarchical data clustering. After the datasets were standardized, multivariate statistical analysis was performed. The R language ropls package was used for principal component analysis (PCA) Single-dimensional statistical analysis included Student's t-test and fold change. The volcano plot was obtained by R software. The standard for differential metabolites was p-value ≤ 0.05 and VIP (variable importance for the projection) ≥ 1.

Organic acid targeted metabolome detection

An organic acid-targeted metabolome was used to analyse the metabolites of nine developmental stages of *H. marmoreus*. There were six biological replicates in this study. Twenty-six kinds of organic acid standard substances were weighed, and the single standard mother liquor was prepared with methanol or water to make a mixed standard. A 30% methanol aqueous solution (containing 0.1% formic acid) was diluted to make a standard working solution, which was stored at 0 °C until LC–MS detection.

Fifty milligrams of tissue sample were weighed into a 2 mL EP tube, and a steel ball was added along with 500 μL of 30% methanol aqueous solution (containing 0.1% formic acid). Then, the samples were ground with a high-throughput tissue grinder at 60 Hz for 120 s and centrifuged at 12000 rpm at 4 °C for 10 min, and the supernatant was added to the detection bottle. The extraction of metabolites was performed according to the methods described in previous research [1]. An ACQUITY UPLC® BEH C18 column (2.1×100 mm, 1.7 μm, Waters Inc., Milford, Massachusetts, USA) was used for chromatographic determination. The sample size was 5 μL, the column temperature was 40 °C, and the mobile phases were A-water (containing 0.1% formic acid) and B-methanol water (containing 0.1% formic acid). Multiple response monitoring (MRM) was used for scanning. Previous research explicitly referred to the chromatographic and mass spectrometric conditions for computer detection [2,3]. Organic acid-targeted metabolome detection was implemented by Shanghai Personal Biotechnology Co. Ltd. The Z score (standard score) is a conversion based on the number of metabolites and is used to measure the number

of metabolites at the same level. The Z score is calculated based on the mean and standard deviation of the control group and is calculated as Z = (x − μ)/σ. X is a specific score, μ is the mean, and σ is the standard deviation.

References

1. Pawlak, M.; Klupczynska, A.; Kokot, Z.J.; Matysiak, J. Extending Metabolomic Studies of Apis mellifera Venom: LC-MS-Based Targeted Analysis of Organic Acids. *Toxins* **2019**, *12*, 14.
2. Langfelder, P.; Horvath, S. WGCNA: An R package for weighted correlation network analysis. *BMC Bioinform.* **2008**, *9*, 559.
3. Liang, Y.; Wang, S.; Zhao, C.; Ma, X.; Zhao, Y.; Shao, J.; Li, Y.; Li, H.; Song, H.; Ma, H.; Li, H.; Zhang, B.; Zhang, L. Transcriptional regulation of bark freezing tolerance in apple (Malus domestica Borkh.). *Hortic. Res.* **2020**, *7*, 205.

Table S1 Primers used for qPCR.

Table S2 KEGG enrichment analysis of the DEGs in LR30 vs. PSR.

Table S3 Correlation analysis of differentially expressed genes (DEGs) and significantly different AAs (SDAs) in OG vs. PS. The associated AAs obtained by correlation analysis of DEGs and SDAs.

Table S4 The associated enzymes obtained by correlation analysis of DEGs and SDAs.

Table S5 KEGG annotation of arginine biosynthesis.

Fig. S1 Unique molecular identifier (UMI) absolute quantitative transcriptome analysis of developmental stages of *H. marmoreus*. (A) Principal component analysis (PCA) of the expressed genes. (B) Heatmap analysis of DEGs. (C) Venn diagram analysis of DEGs. (D) (E) KEGG pathway enrichment of DEGs in OG vs. PS.

Fig. S2 Heatmap analysis of gene expression in the ribosomal pathways.

Fig. S3 KEGG enrichment analysis of the DEGs in LR30_vs_PS.

Fig. S4 KEGG enrichment analysis of the DEGs in OG vs. LR30.

Fig. S5 Expression profile of the amino acid metabolism pathway in low-temperature fruiting after long postripening (LFLP) in *H. marmoreus*. (A) Heatmap analysis of the amino acid metabolism pathway in the LFLP in *H. marmoreus*. (B) The enrichment

analysis of the upregulated DEGs in the postripening growth stages. (C) The enrichment analysis of the upregulated DEGs in reproductive growth stages.

Fig. S6 Targeted profiling of the amino acid metabolome of different developmental stages of *H. marmoreus*. (A) Analysis of total amino acid (AA) content in different developmental stages of *H. marmoreus*. (B) Analysis of each AA content in different developmental stages of *H. marmoreus*.

Fig. S7 PCA of the AAs in the developmental stages of *H. marmoreus.*

Fig. S8 UMI absolute quantitative transcriptome analysis of the developmental stages of *H. marmoreus,* including the substrates of the reproductive growth stage (SRG). (A) PCA of the AAs in the developmental stages of *H. marmoreus.* (B) Heatmap analysis showed the apparent difference in the expression profile in the developmental stages of *H. marmoreus.*

Fig. S9 KEGG enrichment analysis of the DEGs in PSR vs. DSR.

Fig. S10 pH value of substrates in the developmental stages of *H. marmoreus.* The pH values of the substrates from the upper, middle, and lower parts of the cultivation bottle were measured.

Fig. S11 Heatmap analysis of the expression of citrate cycle in the developmental stages of *H. marmoreus.*

Fig. S12 Heatmap analysis of organic acids in the postripening stage (PRS) and SRG.

Fig. S13 WGCNA of the AA-targeted metabolome and transcriptome. (A) Hierarchical cluster dendrogram showing coexpressed modules and a module trait heatmap. Each leaf on the tree represents a gene. Each coloured row indicates a

colour-coded module that contains a group of highly interconnected genes. (B) Heatmap showing the correlation between the modules and AAs. Each row corresponds to a module, whereas each column corresponds to an AA. The correlation coefficient between a given module and an AA is indicated by the colour of the cell at the row-column intersection. Blue and red indicate positive and negative correlations, respectively. (C) The hub genes in the pink modules. (D) Heatmap of the hub genes in the pink module. (E) Enrichment analysis of the genes in the pink modules.

Fig. S14 qPCR analysis of GCN2 and eIF2 in the 4 °C cold stress experiment of *H. marmoreus* mycelia.

Table S1 Primers used for qPCR

Gene	Primer (5' to 3')
Actin-1-F	CCGAGCGGAAGTACTCTGTG
Actin-1-R	ATGCTATCTTGCCTCCAGCC
scaffold1.g953-F	TCTGAAACTGGCGAGCACAT
scaffold1.g953-R	GAGAGCGCGACGATACTTGA
scaffold1.g45-F	GCCGCAAGAAACTCAAGCAA
scaffold1.g45-R	CTGCAAGGTTAGAGGTGGGG
scaffold13.g118-F	CATCCCAACTCCACCCTCAC
scaffold13.g118-R	GGGCCTCCGTAATAGCTTCC
scaffold3.g243-F	AATCGCTCCACCCTCAATCC
scaffold3.g243-R	CGTCTACGCTTCCCGTTGTA

Table S2 KEGG enrichment analysis of the DEGs in LR30 vs. PSR

PathwayID	Pathway	Up_number	Down_number	Total_number	Pvalue
ko03010	Ribosome	26	10	84	2.268E-08
ko04141	Protein processing in endoplasmic reticulum	1	29	88	8.165E-05
ko00500	Starch and sucrose metabolism	19	1	50	0.0001073
ko00040	Pentose and glucuronate interconversions	9	2	25	0.0015746
ko00072	Synthesis and degradation of ketone bodies	1	3	6	0.0097867
ko00511	Other glycan degradation	5	0	9	0.0102632
ko00460	Cyanoamino acid metabolism	7	0	16	0.0119186
ko00520	Amino sugar and nucleotide sugar metabolism	10	8	62	0.0141258
ko00280	Valine, leucine and isoleucine degradation	1	11	37	0.0175387
ko03060	Protein export	0	7	18	0.0242254
ko00900	Terpenoid backbone biosynthesis	3	5	22	0.0249199
ko00650	Butanoate metabolism	1	5	15	0.0315892
ko00910	Nitrogen metabolism	6	1	20	0.0432225
ko00051	Fructose and mannose metabolism	5	3	25	0.0525256
ko00680	Methane metabolism	4	3	22	0.0698304
ko00400	Phenylalanine, tyrosine and tryptophan biosynthesis	2	4	18	0.0743108
ko00020	Citrate cycle (TCA cycle)	1	6	23	0.0861101
ko00360	Phenylalanine metabolism	1	5	19	0.0934619
ko01040	Biosynthesis of unsaturated fatty acids	2	4	19	0.0934619
ko00620	Pyruvate metabolism	5	5	39	0.1205736
ko00600	Sphingolipid metabolism	2	3	16	0.1262228
ko00130	Ubiquinone and other terpenoid-quinone biosynthesis	1	2	8	0.1454938

ko00630	Glyoxylate and dicarboxylate metabolism	3	4	26	0.1464871
ko00052	Galactose metabolism	5	0	18	0.1857701
ko00603	Glycosphingolipid biosynthesis - globo and isoglobo series	2	0	5	0.2072229
ko00010	Glycolysis / Gluconeogenesis	5	4	39	0.2183358
ko00190	Oxidative phosphorylation	1	13	66	0.2368365
ko00640	Propanoate metabolism	1	3	16	0.2924136
ko03018	RNA degradation	1	9	48	0.3073724
ko04213	Longevity regulating pathway - multiple species	2	4	27	0.3166635
ko00220	Arginine biosynthesis	2	2	17	0.3347534
ko00860	Porphyrin and chlorophyll metabolism	0	4	17	0.3347534
ko04146	Peroxisome	2	9	55	0.3447202
ko03450	Non-homologous end-joining	3	0	13	0.3938828
ko00790	Folate biosynthesis	3	0	13	0.3938828
ko00100	Steroid biosynthesis	0	5	24	0.3999146
ko02010	ABC transporters	1	1	8	0.4131598
ko00670	One carbon pool by folate	1	1	8	0.4131598
ko00250	Alanine, aspartate and glutamate metabolism	3	3	30	0.4164648
ko00592	alpha-Linolenic acid metabolism	0	1	3	0.4331871
ko00270	Cysteine and methionine metabolism	2	6	42	0.4400555
ko00230	Purine metabolism	6	2	42	0.4400555
ko00350	Tyrosine metabolism	1	2	14	0.4438349
ko00510	N-Glycan biosynthesis	0	6	31	0.4496102
ko00330	Arginine and proline metabolism	0	6	31	0.4496102
ko00260	Glycine, serine and threonine metabolism	2	5	37	0.4597812
ko00750	Vitamin B6 metabolism	0	1	4	0.5309692
ko00513	Various types of N-glycan biosynthesis	0	4	23	0.5782363
ko00062	Fatty acid elongation	0	1	5	0.6119058
ko00430	Taurine and hypotaurine metabolism	1	0	5	0.6119058
ko00071	Fatty acid degradation	1	3	24	0.6139007
ko00030	Pentose phosphate pathway	3	1	24	0.6139007

ko00340	Histidine metabolism	2	2	24	0.6139007

Table S3

CompoundID	Description	Number	Pathway
C00025	Glu	30	33
C00073	Met	13	6
C00037	Gly	11	15
C00047	Lys	11	7
C00064	Gln	10	15
C00049	Asp	9	17
C00334	GABA	7	11
C00065	Ser	5	14
C00188	Thr	5	8
C00135	His	5	6
C00407	Ile	4	9
C00123	Leu	4	8
C00041	Ala	3	11
C00152	Asn	3	6
C00062	Arg	3	12

Table S4

EC	Annotation	Number	Associated compoundID
2.1.1.-	Methyltransferase	10	C00073;C00135
2.3.1.-	Acetyltransferase	8	C00037;C00047
2.6.1.42	Aminotransferase	8	C00183;C00407;C00123;C00025
6.3.5.4	Asparagine synthase	8	C00152;C00049;C00064;C00025
1.2.1.3	Aldehyde dehydrogenase	6	C00334
3.5.1.-	Amidohydrolase	6	C00047
4.1.3.27	Anthranilate synthase	4	C00064;C00025
6.3.5.1	Glutamine-hydrolysing	4	C00064;C00025
4.1.1.15	Glutamate decarboxylase	3	C00334;C00049;C00025
3.4.-.-	Assembly-enhancing protease	3	C00188
6.1.1.12	Aspartyl-tRNA synthetase	3	C00049
2.6.1.1	Aminotransferase	3	C00049;C00025;C00082

Table S5

ID	KO	EC	Definition
scaffold5.g62	K01940	6.3.4.5	Argininosuccinate synthase
scaffold9.g163	K01476	3.5.3.1	Arginase
scaffold10.g18	K00611	2.1.3.3	Ornithine carbamoyltransferase
scaffold33.g38	K00814	2.6.1.2	Alanine transaminase
scaffold3.g399	K15371	1.4.1.2	Glutamate dehydrogenase
scaffold24.g62	K01941	6.3.4.6	Urea carboxylase
scaffold1.g1037	K01968	6.4.1.4	3-methylcrotonyl-coa carboxylase alpha subunit
scaffold5.g166	K01958	6.4.1.1	Pyruvate carboxylase
scaffold5.g358	K11262	6.4.1.2	Acetyl-coa carboxylase / biotin carboxylase 1
scaffold4.g68	K14454	2.6.1.1	Aspartate aminotransferase, cytoplasmic
scaffold1.g592	K14455	2.6.1.1	Aspartate aminotransferase, mitochondrial
scaffold2.g414	K01915	6.3.1.2	Glutamine synthetase
scaffold2.g415	K01915	6.3.1.2	Glutamine synthetase
scaffold4.g71	K01755	4.3.2.1	Argininosuccinate lyase
scaffold22.g107	K00262	1.4.1.4	Glutamate dehydrogenase (NADP+)
scaffold18.g129	K12659	2.7.2.8	N-acetyl-gamma-glutamyl-phosphate reductase / acetylglutamate kinase
scaffold2.g682	K00818	2.6.1.11	Acetylornithine aminotransferase
scaffold15.g113	K00620	2.3.1.1	Glutamate n-acetyltransferase / amino-acid n-acetyltransferase

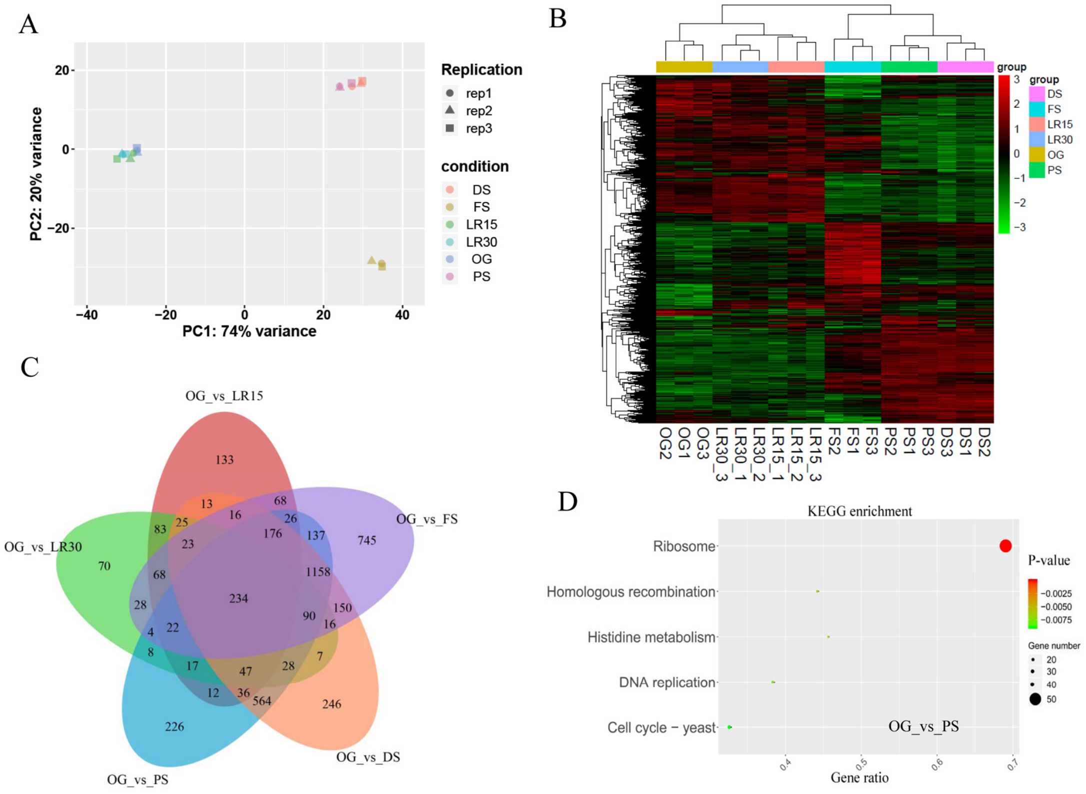
A
B
C
D
Replication
rep1
rep2
rep3
condition
DS
FS
LR15
LR30
OG
PS
PC1: 74% variance
PC2: 20% variance
group
OG_vs_LR15
OG_vs_FS
OG_vs_LR30
OG_vs_PS
OG_vs_DS
KEGG enrichment
Ribosome
Homologous recombination
Histidine metabolism
DNA replication
Cell cycle − yeast
P-value
Gene number
Gene ratio

Fig. S1

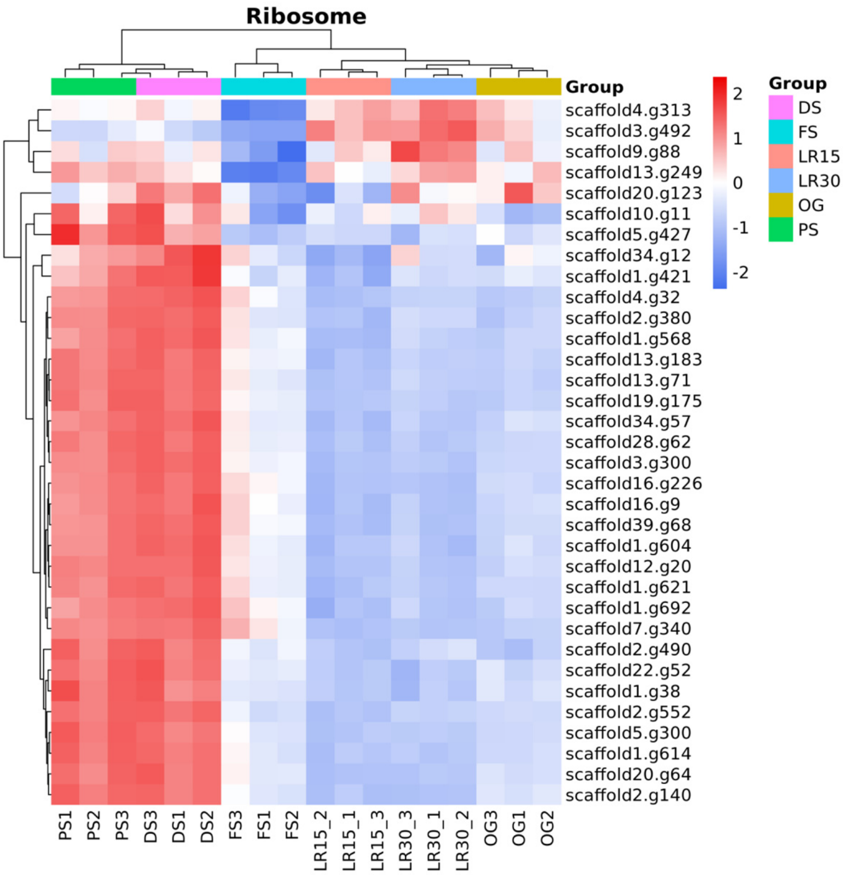

Ribosome
Group
scaffold4.g313
scaffold3.g492
scaffold9.g88
scaffold13.g249
scaffold20.g123
scaffold10.g11
scaffold5.g427
scaffold34.g12
scaffold1.g421
scaffold4.g32
scaffold2.g380
scaffold1.g568
scaffold13.g183
scaffold13.g71
scaffold19.g175
scaffold34.g57
scaffold28.g62
scaffold3.g300
scaffold16.g226
scaffold16.g9
scaffold39.g68
scaffold1.g604
scaffold12.g20
scaffold1.g621
scaffold1.g692
scaffold7.g340
scaffold2.g490
scaffold22.g52
scaffold1.g38
scaffold2.g552
scaffold5.g300
scaffold1.g614
scaffold20.g64
scaffold2.g140
PS1
PS2
PS3
DS3
DS1
DS2
FS3
FS1
FS2
LR15_2
LR15_1
LR15_3
LR30_3
LR30_1
LR30_2
OG3
OG1
OG2
2
1
0
-1
-2
Group
DS
FS
LR15
LR30
OG
PS

Fig. S2

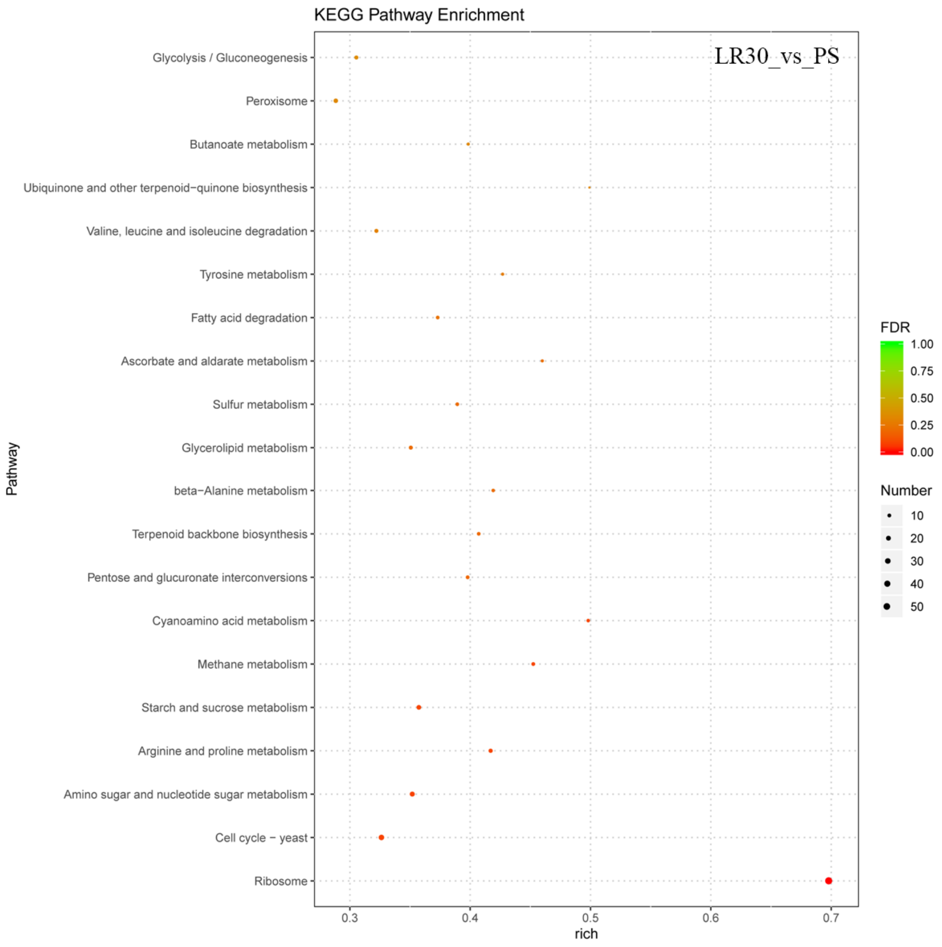

KEGG Pathway Enrichment
LR30_vs_PS
Glycolysis / Gluconeogenesis
Peroxisome
Butanoate metabolism
Ubiquinone and other terpenoid−quinone biosynthesis
Valine, leucine and isoleucine degradation
Tyrosine metabolism
Fatty acid degradation
Ascorbate and aldarate metabolism
Sulfur metabolism
Glycerolipid metabolism
beta−Alanine metabolism
Terpenoid backbone biosynthesis
Pentose and glucuronate interconversions
Cyanoamino acid metabolism
Methane metabolism
Starch and sucrose metabolism
Arginine and proline metabolism
Amino sugar and nucleotide sugar metabolism
Cell cycle − yeast
Ribosome
Pathway
0.3
0.4
0.5
0.6
0.7
rich
FDR
1.00
0.75
0.50
0.25
0.00
Number
10
20
30
40
50

Fig. S3

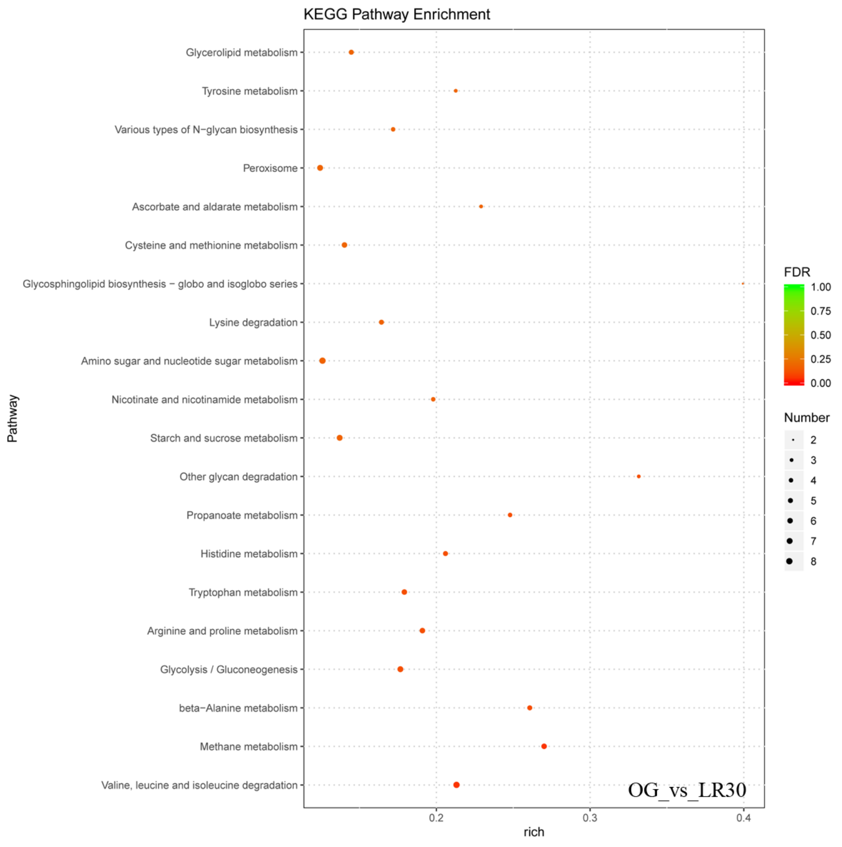
KEGG Pathway Enrichment
Glycerolipid metabolism
Tyrosine metabolism
Various types of N−glycan biosynthesis
Peroxisome
Ascorbate and aldarate metabolism
Cysteine and methionine metabolism
Glycosphingolipid biosynthesis − globo and isoglobo series
Lysine degradation
Amino sugar and nucleotide sugar metabolism
Nicotinate and nicotinamide metabolism
Starch and sucrose metabolism
Other glycan degradation
Propanoate metabolism
Histidine metabolism
Tryptophan metabolism
Arginine and proline metabolism
Glycolysis / Gluconeogenesis
beta−Alanine metabolism
Methane metabolism
Valine, leucine and isoleucine degradation
Pathway
0.2
0.3
0.4
rich
OG_vs_LR30
FDR
1.00
0.75
0.50
0.25
0.00
Number
2
3
4
5
6
7
8

Fig. S4

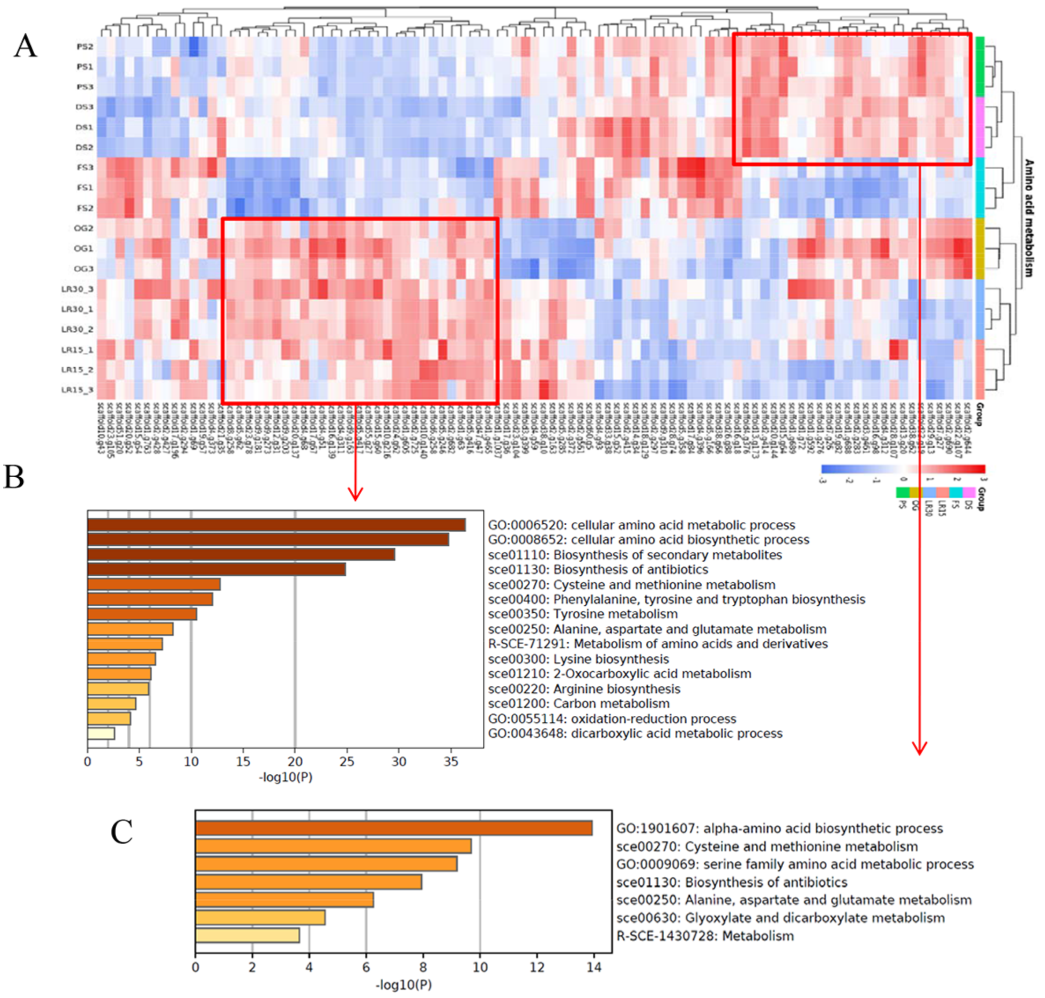
A
PS2
PS1
PS3
DS3
DS1
DS2
FS3
FS1
FS2
OG2
OG1
OG3
LR30_3
LR30_1
LR30_2
LR15_1
LR15_2
LR15_3
Amino acid metabolism
Group
PS
OG
LR30
LR15
FS
DS
B
GO:0006520: cellular amino acid metabolic process
GO:0008652: cellular amino acid biosynthetic process
sce01110: Biosynthesis of secondary metabolites
sce01130: Biosynthesis of antibiotics
sce00270: Cysteine and methionine metabolism
sce00400: Phenylalanine, tyrosine and tryptophan biosynthesis
sce00350: Tyrosine metabolism
sce00250: Alanine, aspartate and glutamate metabolism
R-SCE-71291: Metabolism of amino acids and derivatives
sce00300: Lysine biosynthesis
sce01210: 2-Oxocarboxylic acid metabolism
sce00220: Arginine biosynthesis
sce01200: Carbon metabolism
GO:0055114: oxidation-reduction process
GO:0043648: dicarboxylic acid metabolic process
0 5 10 15 20 25 30 35
-log10(P)
C
GO:1901607: alpha-amino acid biosynthetic process
sce00270: Cysteine and methionine metabolism
GO:0009069: serine family amino acid metabolic process
sce01130: Biosynthesis of antibiotics
sce00250: Alanine, aspartate and glutamate metabolism
sce00630: Glyoxylate and dicarboxylate metabolism
R-SCE-1430728: Metabolism
0 2 4 6 8 10 12 14
-log10(P)

Fig. S5

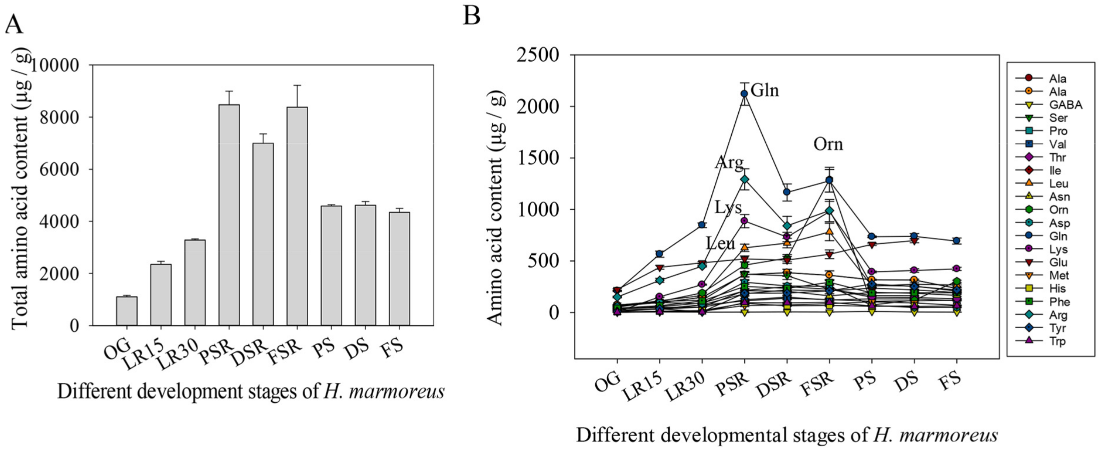
A
Total amino acid content (μg / g)
10000
8000
6000
4000
2000
0
OG
LR15
LR30
PSR
DSR
FSR
PS
DS
FS
Different development stages of H. marmoreus
B
Amino acid content (μg / g)
2500
2000
1500
1000
500
0
Gln
Orn
Arg
Lys
Leu
OG
LR15
LR30
PSR
DSR
FSR
PS
DS
FS
Different developmental stages of H. marmoreus
Ala
Ala
GABA
Ser
Pro
Val
Thr
Ile
Leu
Asn
Orn
Asp
Gln
Lys
Glu
Met
His
Phe
Arg
Tyr
Trp

Fig. S6

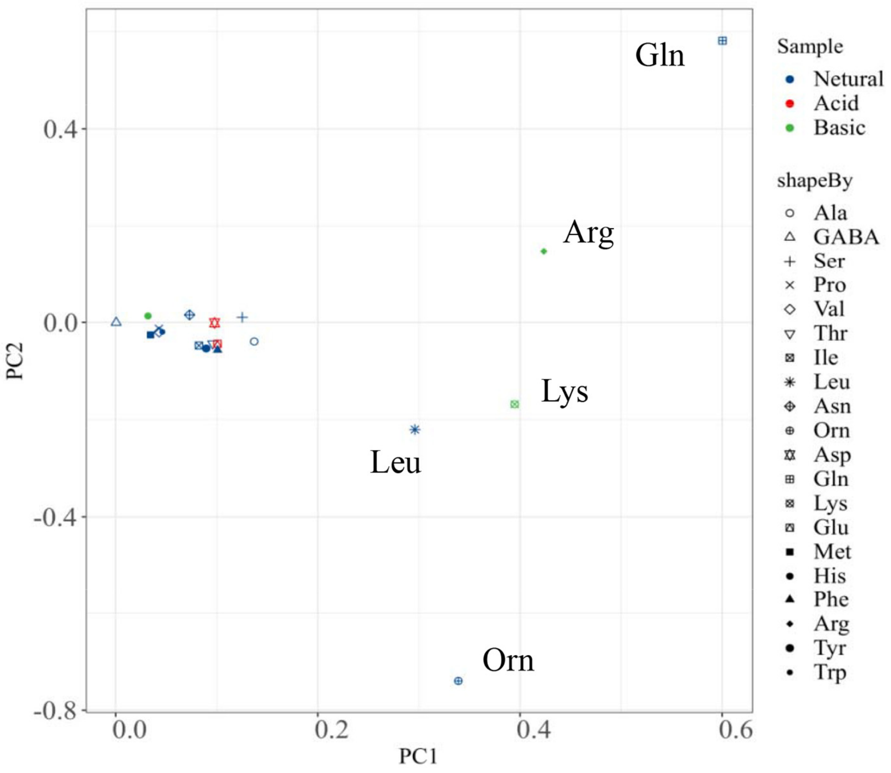

Gln
Arg
Lys
Leu
Orn
0.4
0.0
-0.4
-0.8
0.0
0.2
0.4
0.6
PC1
PC2
Sample
Netural
Acid
Basic
shapeBy
Ala
GABA
Ser
Pro
Val
Thr
Ile
Leu
Asn
Orn
Asp
Gln
Lys
Glu
Met
His
Phe
Arg
Tyr
Trp

Fig. S7

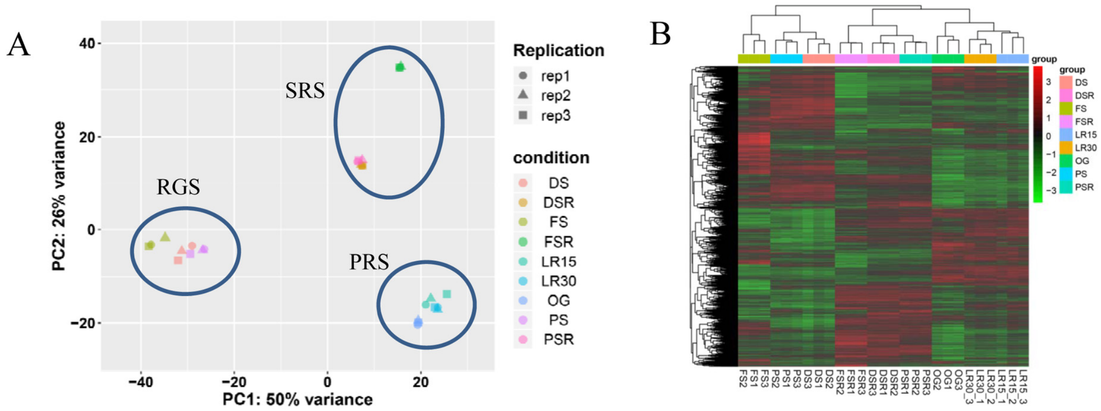
A
B
SRS
RGS
PRS
PC2: 26% variance
PC1: 50% variance
Replication
rep1
rep2
rep3
condition
DS
DSR
FS
FSR
LR15
LR30
OG
PS
PSR
group

Fig. S8

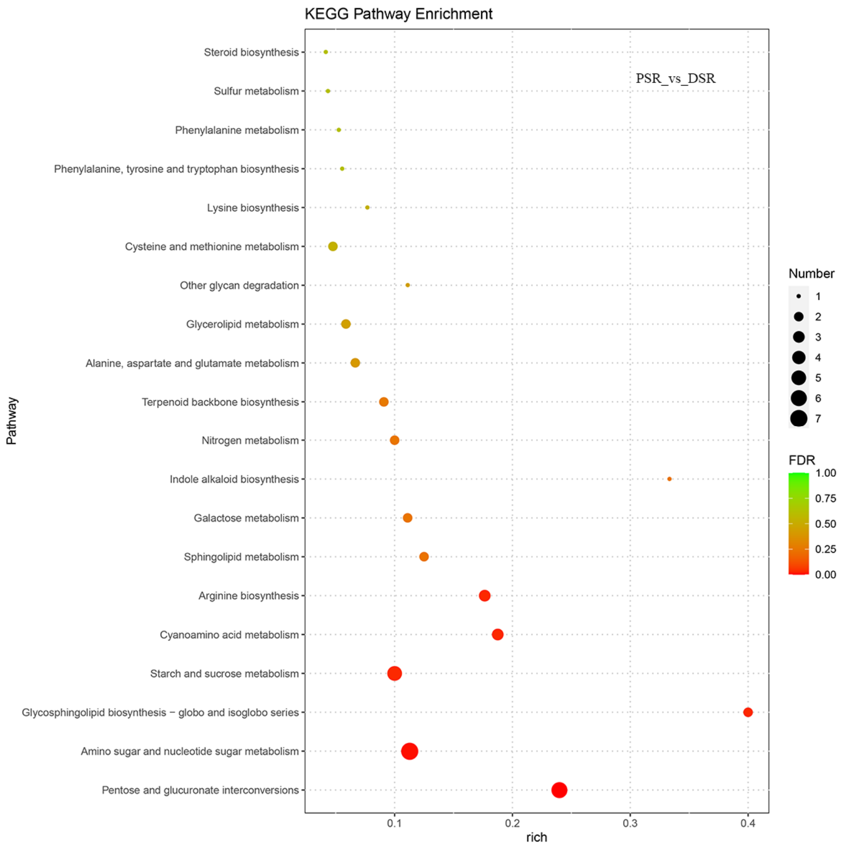

KEGG Pathway Enrichment
PSR_vs_DSR
Steroid biosynthesis
Sulfur metabolism
Phenylalanine metabolism
Phenylalanine, tyrosine and tryptophan biosynthesis
Lysine biosynthesis
Cysteine and methionine metabolism
Other glycan degradation
Glycerolipid metabolism
Alanine, aspartate and glutamate metabolism
Terpenoid backbone biosynthesis
Nitrogen metabolism
Indole alkaloid biosynthesis
Galactose metabolism
Sphingolipid metabolism
Arginine biosynthesis
Cyanoamino acid metabolism
Starch and sucrose metabolism
Glycosphingolipid biosynthesis − globo and isoglobo series
Amino sugar and nucleotide sugar metabolism
Pentose and glucuronate interconversions
Pathway
0.1
0.2
0.3
0.4
rich
Number
1
2
3
4
5
6
7
FDR
1.00
0.75
0.50
0.25
0.00

Fig. S9

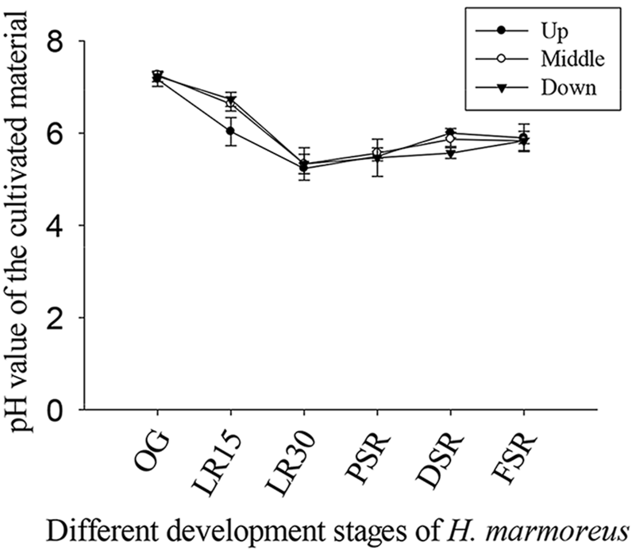
pH value of the cultivated material
8
6
4
2
0
Up
Middle
Down
OG
LR15
LR30
PSR
DSR
FSR
Different development stages of H. marmoreus

Fig. S10

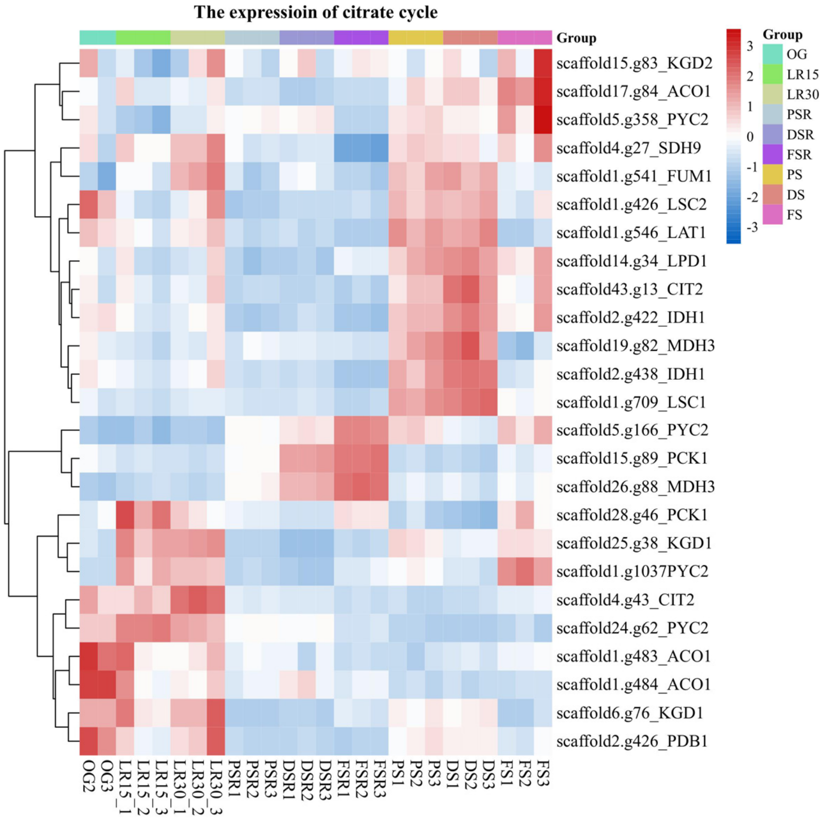
The expressioin of citrate cycle
Group
scaffold15.g83_KGD2
scaffold17.g84_ACO1
scaffold5.g358_PYC2
scaffold4.g27_SDH9
scaffold1.g541_FUM1
scaffold1.g426_LSC2
scaffold1.g546_LAT1
scaffold14.g34_LPD1
scaffold43.g13_CIT2
scaffold2.g422_IDH1
scaffold19.g82_MDH3
scaffold2.g438_IDH1
scaffold1.g709_LSC1
scaffold5.g166_PYC2
scaffold15.g89_PCK1
scaffold26.g88_MDH3
scaffold28.g46_PCK1
scaffold25.g38_KGD1
scaffold1.g1037PYC2
scaffold4.g43_CIT2
scaffold24.g62_PYC2
scaffold1.g483_ACO1
scaffold1.g484_ACO1
scaffold6.g76_KGD1
scaffold2.g426_PDB1
OG2
OG3
LR15_1
LR15_2
LR15_3
LR30_1
LR30_2
LR30_3
PSR1
PSR2
PSR3
DSR1
DSR2
DSR3
FSR1
FSR2
FSR3
PS1
PS2
PS3
DS1
DS2
DS3
FS1
FS2
FS3
3
2
1
0
-1
-2
-3
Group
OG
LR15
LR30
PSR
DSR
FSR
PS
DS
FS

Fig. S11

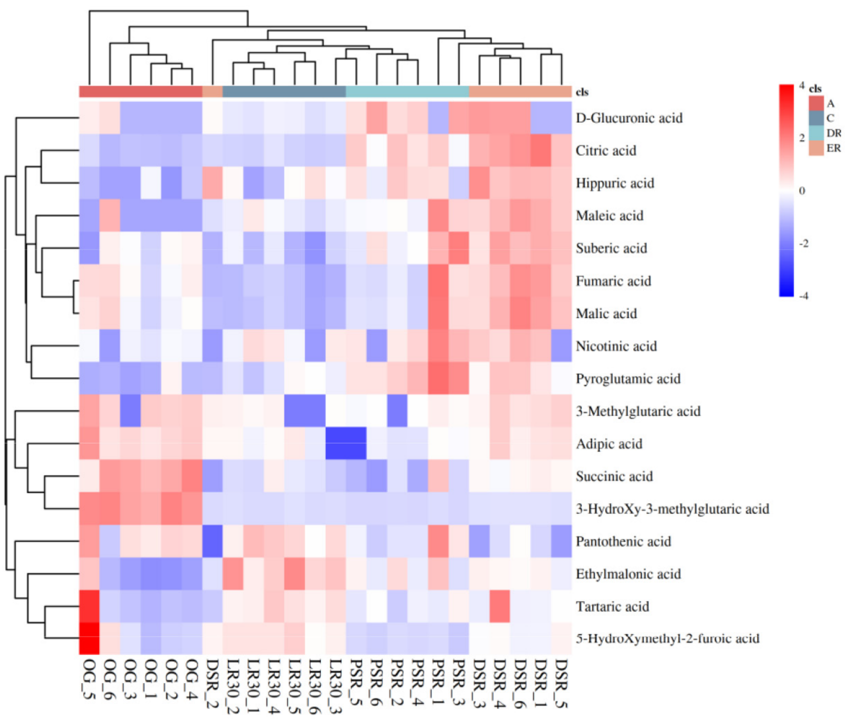

cls
A
C
DR
ER
4
2
0
-2
-4
D-Glucuronic acid
Citric acid
Hippuric acid
Maleic acid
Suberic acid
Fumaric acid
Malic acid
Nicotinic acid
Pyroglutamic acid
3-Methylglutaric acid
Adipic acid
Succinic acid
3-HydroXy-3-methylglutaric acid
Pantothenic acid
Ethylmalonic acid
Tartaric acid
5-HydroXymethyl-2-furoic acid
OG_5
OG_6
OG_3
OG_1
OG_2
OG_4
DSR_2
LR30_2
LR30_1
LR30_4
LR30_5
LR30_6
LR30_3
PSR_5
PSR_6
PSR_2
PSR_4
PSR_1
PSR_3
DSR_3
DSR_4
DSR_6
DSR_1
DSR_5

Fig. S12

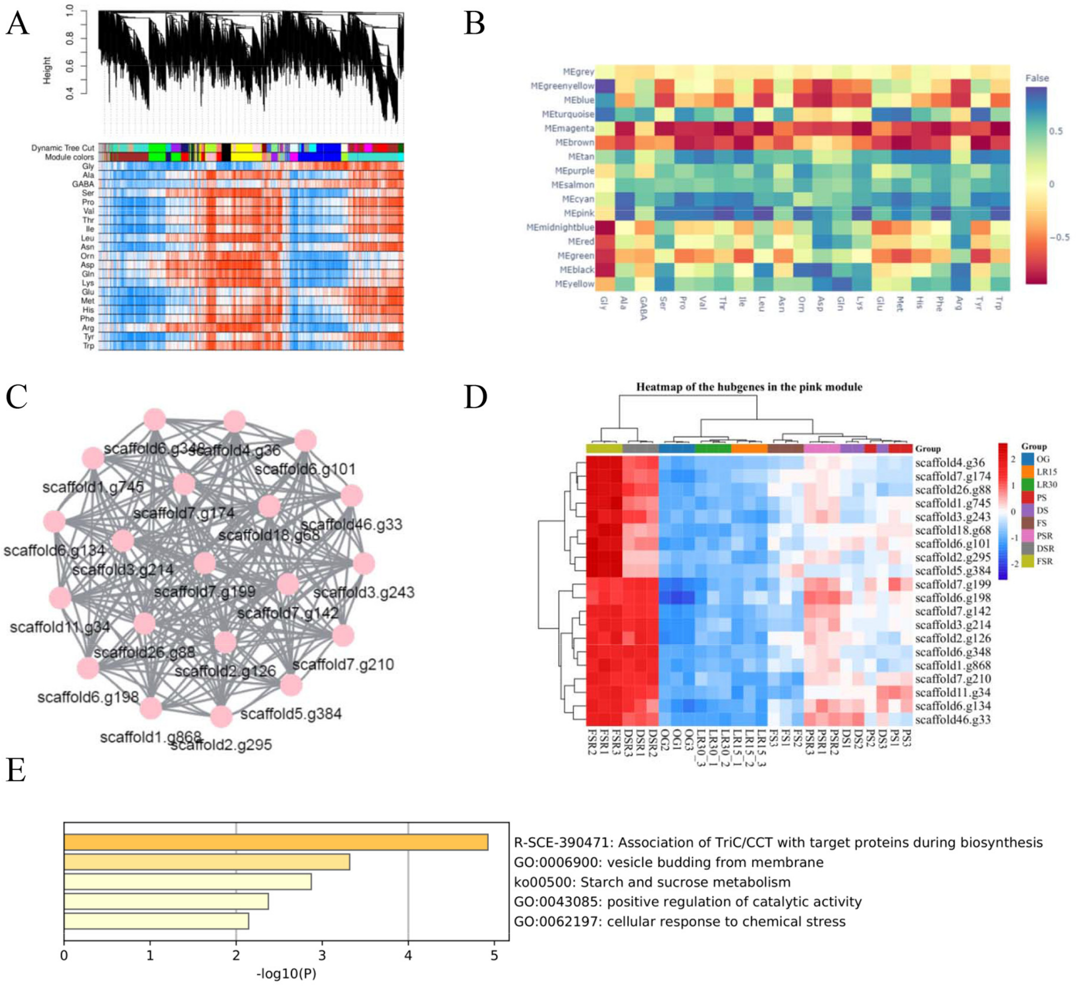
A
Height
1.0
0.8
0.6
0.4
Dynamic Tree Cut
Module colors
Gly
Ala
GABA
Ser
Pro
Val
Thr
Ile
Leu
Asn
Orn
Asp
Gln
Lys
Glu
Met
His
Phe
Arg
Tyr
Trp
B
MEgrey
MEgreenyellow
MEblue
MEturquoise
MEmagenta
MEbrown
MEtan
MEpurple
MEsalmon
MEcyan
MEpink
MEmidnightblue
MEred
MEgreen
MEblack
MEyellow
False
0.5
0
−0.5
C
scaffold6.g348
scaffold4.g36
scaffold6.g101
scaffold1.g745
scaffold7.g174
scaffold18.g68
scaffold46.g33
scaffold6.g134
scaffold3.g214
scaffold7.g199
scaffold3.g243
scaffold7.g142
scaffold11.g34
scaffold26.g88
scaffold2.g126
scaffold7.g210
scaffold6.g198
scaffold5.g384
scaffold1.g868
scaffold2.g295
D
Heatmap of the hubgenes in the pink module
Group
OG
LR15
LR30
PS
DS
FS
PSR
DSR
FSR
scaffold4.g36
scaffold7.g174
scaffold26.g88
scaffold1.g745
scaffold3.g243
scaffold18.g68
scaffold6.g101
scaffold2.g295
scaffold5.g384
scaffold7.g199
scaffold6.g198
scaffold7.g142
scaffold3.g214
scaffold2.g126
scaffold6.g348
scaffold1.g868
scaffold7.g210
scaffold11.g34
scaffold6.g134
scaffold46.g33
FSR2
FSR1
FSR3
DSR3
DSR1
DSR2
OG2
OG1
OG3
LR30_3
LR30_1
LR30_2
LR15_1
LR15_2
LR15_3
FS3
FS1
FS2
PSR3
PSR1
PSR2
DS1
DS2
PS2
DS3
PS1
PS3
E
R-SCE-390471: Association of TriC/CCT with target proteins during biosynthesis
GO:0006900: vesicle budding from membrane
ko00500: Starch and sucrose metabolism
GO:0043085: positive regulation of catalytic activity
GO:0062197: cellular response to chemical stress
0
1
2
3
4
5
-log10(P)

Fig. S13

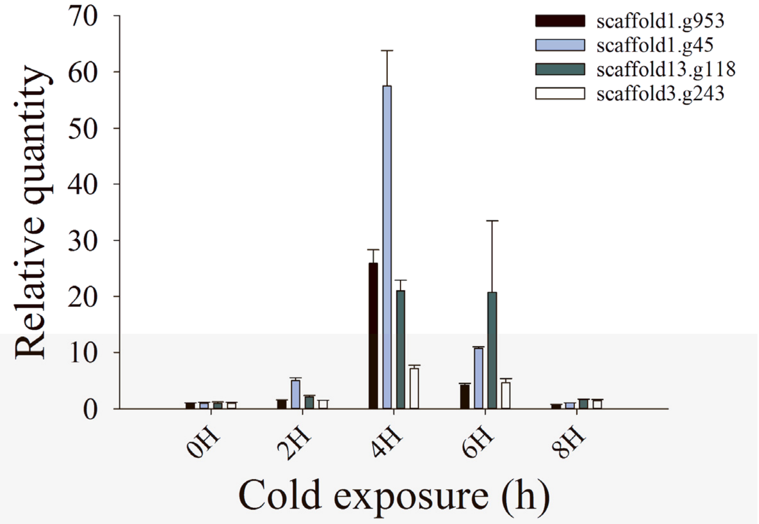

scaffold1.g953
scaffold1.g45
scaffold13.g118
scaffold3.g243
Relative quantity
70
60
50
40
30
20
10
0
0H
2H
4H
6H
8H
Cold exposure (h)

Fig. S14
